# Paleoreconstructions of ciliate communities reveal long-term ecological changes in temperate lakes

**DOI:** 10.1038/s41598-022-12041-7

**Published:** 2022-05-12

**Authors:** Cécilia Barouillet, Valentin Vasselon, François Keck, Laurent Millet, David Etienne, Didier Galop, Damien Rius, Isabelle Domaizon

**Affiliations:** 1 INRAE, Université Savoie Mont Blanc, CARRTEL, 74200 Thonon-les-Bains, France; 2 Pole R&D ECLA, CARRTEL, 74200 Thonon-les-Bains, France; 3 OFB, Site INRAE UMR CARRTEL, 74200 Thonon-les-Bains, France; 4 CNRS, Chrono Environnement, 25000 Besançon, France; 5 Université Savoie Mont Blanc, INRAE, CARRTEL, 73370 Le Bourget du Lac, France; 6 GEODE UMR 5602 CNRS, Université de Toulouse, 31058 Toulouse, France; 7 Labex DRIIHM, OHM Pyrénées, CNRS/INEE, Toulouse, France

**Keywords:** Freshwater ecology, Molecular ecology, Palaeoecology, Ecology, Limnology

## Abstract

Ciliates are unicellular heterotrophic organisms that play a key role in aquatic planktonic and benthic food webs. Advances in sedimentary DNA (sed-DNA) analysis offer the possibility to integrate these bioindicators in paleoenvironmental reconstructions. In this study, we used the top–bottom paleolimnological approach and metabarcoding techniques applied to sed-DNA to compare the recent and past (i.e. prior to major anthropogenic impacts) ciliate communities of 48 lakes located along an elevation gradient. Our results show an overall decline in the β-diversity in recent time, especially in lowland lakes, which are more strongly exposed to local human pressures. Analyses of the functional groups indicate important restructuration of the food web, including the recent increase in mixotrophs. Moreover, changes in the benthic ciliates were consistent with the widespread increase in deep water anoxia. Our results provided evidence that sed-DNA can uncover information about past ciliate communities on a wide variety of lakes. Overall, our study demonstrates the potential of using ciliates as new paleoindicators, integrating information from the pelagic to the benthic zones, and providing valuable insights into ecosystem functioning through a trait-based functional community approach. As paleoindicator, they thus offer a more holistic view on the long-term changes of aquatic ecosystems.

## Introduction

Lakes are largely recognized as integrators and sentinels of environmental changes^1^. Pressures from anthropogenic activities have largely increased in magnitude since the mid-twentieth century, a period that has also been referred to as the Great Acceleration^2,3^. More specifically, climate change and the human alteration of the landscapes can have a profound impact on the physical and chemical characteristics of lakes^4,5^, thereby influencing the communities living in and depending on these ecosystems^6^. While challenging, assessing the biological response to environmental changes over large geographical scales can provide important insight into the vulnerability of lakes to anthropogenic and climate forcing. The top–bottom paleolimnological approach allows such assessment for aquatic communities through the comparison of sedimentary archives of recent (i.e. top samples) and past communities (i.e. bottom sample)^7^. This comparative approach is quite powerful as it can be applied on large geographical scales and brings important insight into reference conditions (i.e. prior to major anthropogenic influences), thereby providing an assessment of the amplitude of change^8,9^

Although the diagenesis and archiving of DNA in sediments through time is often discussed in literature^10^, previous studies have demonstrated that the genetic information of the microorganisms living in the water column is archived in the sediments^11–13^. Over the last decades, the development of molecular biology techniques to target DNA molecules preserved in lake sediments (sed-DNA) largely expanded the field of paleolimnology^14^. Applying molecular biology tools in paleolimnological investigations have allowed to reconstruct historical freshwater biodiversity^15^. Through the implementation of new paleo-indicators, sed-DNA provides access to information about the long-term dynamic of organisms for which morphological remains are not preserved^16,17^, as well as overlooked communities. This includes microorganisms^18,19^, which represent the largest source of biodiversity and ecological functions. As such, sed-DNA offers new opportunities to reconstruct a more holistic view of the long-term biological dynamic of lakes^18,20^ and integrate microorganisms in the debates concerning the response of biodiversity to ongoing environmental changes^19^.

Ciliates are unicellular microorganisms occupying diverse ecological niches and are widely distributed^21^. These protists display a large functional diversity, acting as predators of bacteria and other protists, including algae, as well as small metazoans^22^, while mixotrophic ciliates can significantly enhance primary production^23,24^. Altogether, they play a key role in aquatic pelagic and benthic food webs, especially in the transfer of energy and nutrient cycling from the microbial loop to the metazooplankton^21,25^. Long-term reconstruction of the microbial eukaryote diversity of large peri-alpine lakes reported a strong relative contribution of the ciliate communities to the total abundance of microeukaryotic communities^13^. These studies also demonstrated that ciliates are sensitive to changes in phosphorus concentration. Although the ciliates are good indicators of environmental changes^25,26^, they are seldom used in routine limnological surveys. Indeed, the analysis of ciliate communities through microscopic-based approach and morphospecies identifications is challenging^27^, mostly because the monophyletic group of ciliates includes very different organisms that display diverse and complex life cycles^28^. Recent advances in molecular biology and the development of more robust taxonomic libraries continue to strengthen eDNA and sed-DNA techniques^28,29^, thereby allowing to include these understudied groups and useful indicators for a more holistic ecological diagnosis.

**Figure 1:**
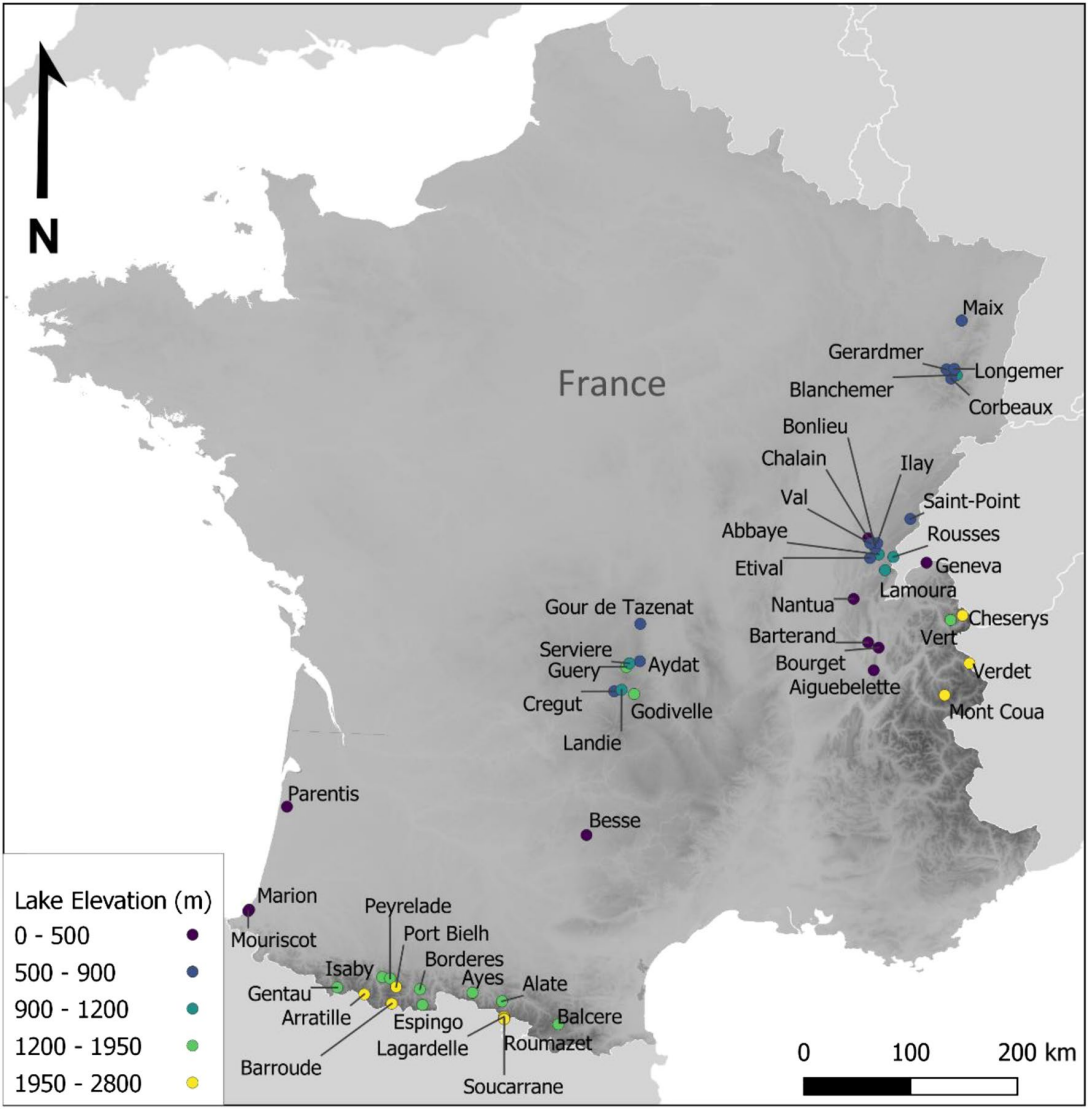
Location of the 48 studied lakes. The colours correspond to associated elevation range (cf. legend), the grey gradient illustrates the elevation terrain (DEM; source: https://srtm.csi.cgiar.org/) within the geopolitical boarders of France.

In the present study, we combine metabarcoding methods and the top–bottom paleolimnological approach to reconstruct recent and past ciliate communities of 48 temperate lakes (Fig. 1). Drastic changes in the composition of the microeukaryotic communities of these lakes were previously observed as a response to the Great Acceleration^18^. More specifically, a significant increase in phototrophic and mixotrophic communities was recorded, which was consistent with the global enhancement of primary productivity. Focusing on these same lakes, we aimed at exploring how the heterotrophic and mixotrophic groups of ciliates responded to these recent changes by (1) assessing the amplitude of changes in the ciliate communities across a wide variety of lakes and (2) evaluating the potential of using the ciliate as indicators of functional and biological changes in paleo-environmental reconstructions.

## Results

### Ciliates metabarcoding analyses

The sequencing resulted in a total number of 2,746,319 DNA reads with an average of 28,650 reads per sample. After the filtering steps, 1,745,532 reads were retained and clustered into 2446 OTUs. Detailed information about the effect of bioinformatics treatments on the DNA reads are accessible in the supplementary information along with a summary of the resulting total number of OTU and number of reads taxonomically assigned and associated to a functional trait (Supplemental Tables S1, S2).

### Ciliates community diversity

**Figure 2:**
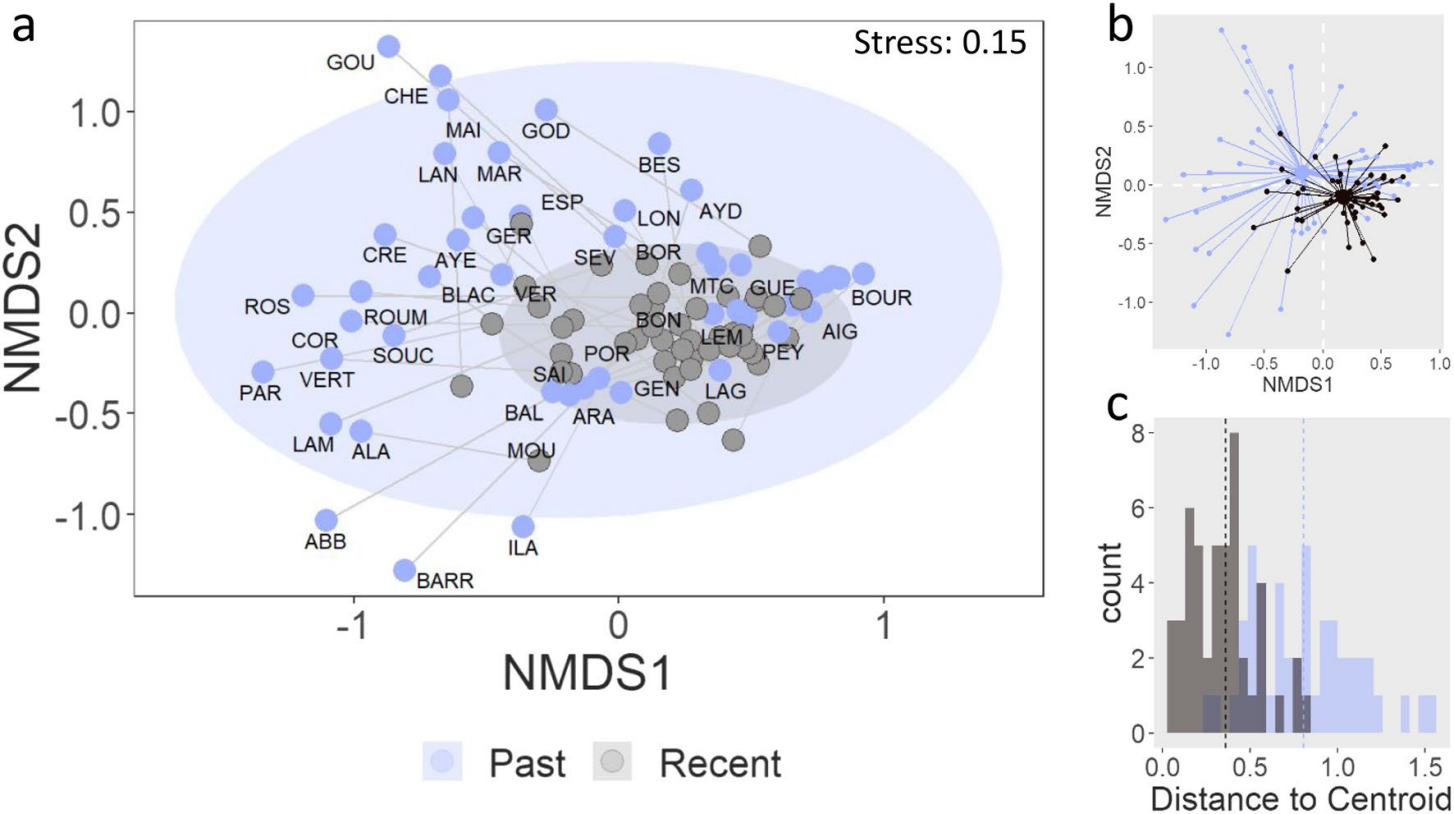
(**a**) NMDS of the community composition of the recent (grey dots) and past (purple dots) samples with the 95% confidence ellipses represented for each group. The past samples are labelled with their corresponding lake code (cf. Table S3), the grey lines connect recent and past samples from the same lake. Note: some labels are missing to avoid overlapping labels. (**b**) Spider plot showing the location of the geometric median of each group (i.e. past and recent) and illustrating the distance of the recent and past samples related to the geometric median of their respective group. (**c**) Distribution of the distances between samples and group geometric median (i.e. centroid) for recent and past samples, the geometric median of each group is indicated by the dotted line.

The NMDS based on the Bray–Curtis distance (i.e. a measure of the amplitude of change in the β-diversity of the ciliates at the community level between the past and recent samples) showed that the overall dispersion of the recent samples (i.e. top of the core, corresponding to recent time ~ 2000 C.E.) and past samples (i.e. bottom samples of the core, corresponding to pre-industrial time prior to the 1850s) differed, with the distance between the samples and their geometric median being much more variable and greater for the bottom samples (Fig. 2).

The average distance between the samples and their geometric median was significantly different between the past 0.79 and recent samples 0.35 (Wilcoxon test, *p* < 0.01). However, there was no large displacement of the geometric median relative to each other as illustrated by the overlapping confidence ellipses of the two groups (Fig. 2a). This pattern was even more pronounced on the NMDS calculated from the OTU table (Supplemental Fig. S1).

**Figure 3:**
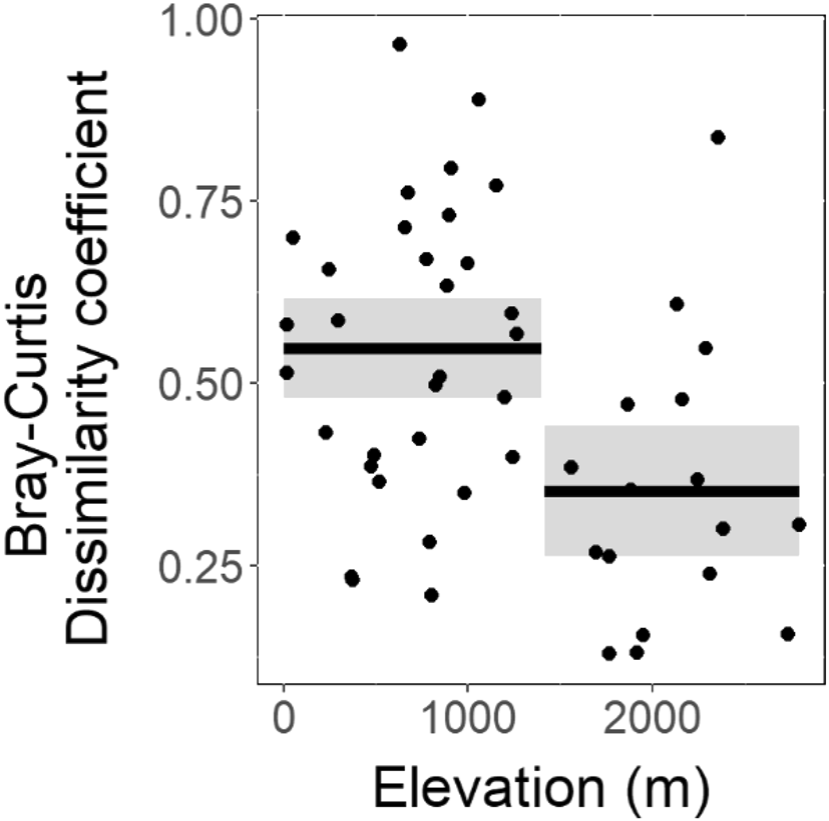
Distribution of the Bray–Curtis dissimilarity coefficients between recent and past samples (black dot) according to lake elevation (m). The fitted regression tree model (n = 48 lakes) identified a split at 1400 m and is represented by black lines (mean values). Gray shading represents the 95% confidence intervals around the means.

The regression tree analysis identified a significant split in the dataset at an elevation of 1400 m, with a Bray–Curtis dissimilarity coefficient averaging 0.37 for lakes situated above 1400 m in elevation while lakes situated under 1400 m in elevation displayed an average of 0.57 (Fig. 3).

**Figure 4:**
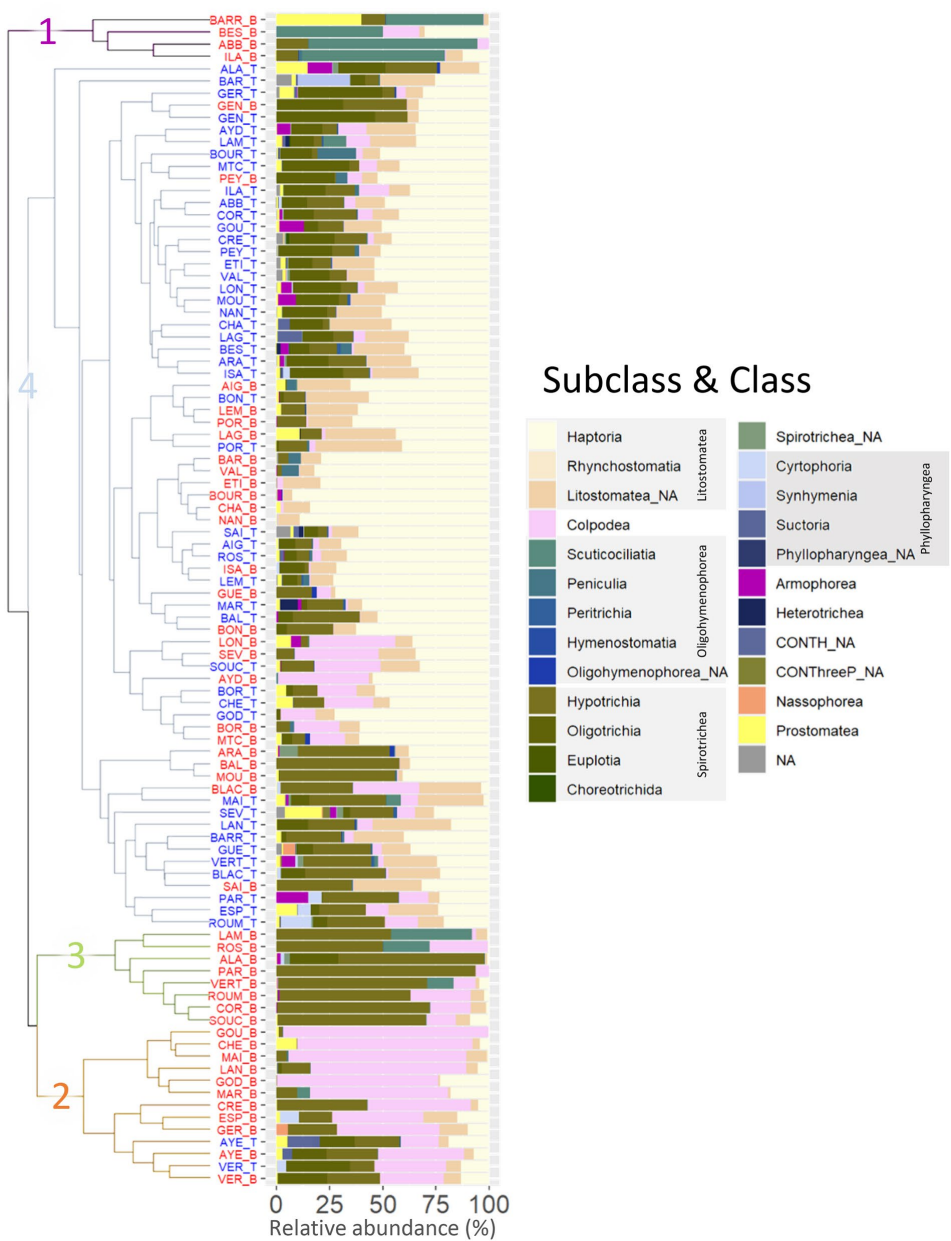
Dendrogram illustrating the results from the hierarchical cluster analysis. On the right side of the figure, the ciliate community composition is represented as the relative abundance of classes and subclasses (% of DNA reads). The sample labels correspond to the lake code (cf. Table S3) followed by “_T” or “_B” indicating the recent (i.e. “top”, in blue) or past (i.e. “bottom”, in red) samples respectively. In the legend, *ClassName*_NA was used whenever the Subclass could not be assigned.

The hierarchical analysis identified four clusters according to their community composition, separating most past samples from the recent samples (Fig. 4). More specifically, the clusters 1, 2 and 3 were only composed of past samples except for the cluster 2, which contained the present and past samples of two lakes. The samples of cluster 1 were dominated by the Oligohymenophorea (Class) Scuticociliatia (Subclass), samples of cluster 2 by Colpodea (mainly Cyrtolophosidida), while samples of cluster 3 were dominated by Spirotrichea Hypotrichia (Fig. 4). The ciliate communities of the samples from the fourth cluster were dominated by the Class Litostomatea, subclass Haptoria. Although the fourth cluster regrouped past and recent samples, sub-clusters indicated a clear separation of the recent samples from the past samples. When dividing the dataset into regional subgroups, the hierarchical clustering analyses also separate the recent samples from the past samples (Fig. S2). This is with the exception of the high elevation lakes located above 1400 m from the Alps and the Pyrenees. Across all regions, the ciliate community of the recent samples tend to be dominated by the Class Litostomatea, subclass Haptoria. In contrast, ciliate community of the past samples differs between regions. More specifically, lakes from the Vosges and Massif Central were previously dominated by Colpodea, lakes from the Jura and the Alps were previously dominated by Scuticociliata or Litostomatea. The SIMPER analysis applied on the subclass level indicated that the increase of the Haptoria, the Oligotrichia and Armophorea explained 26%, 11% and 2% respectively of the Bray–Curtis dissimilarity between the recent and past samples, while the decline of Colpodea, Hypotrichia, Scuticociliatia and Prostomatea explained 19%, 18%, 6% and 3% of the dissimilarity. The differential abundance analysis (DESeq2) provided information about the intensity of the changes between the recent and past samples^30^. The DESeq2 applied at the Genus level, indicated a significant increase of five genera and families of the subclass Haptoria, two genera and families of the subclass Oligotrichia and the genus Metopus (class Armophorea), as well as a significant increase of the Peritrichia and Suctoria (Supplemental Fig. S3). The DESeq2 analysis also indicated a significant decline of *Urotricha* a genus of the class Prostomatea, two Scuticociliatia including *Cyclidium*, five genera and families associated to the class Colpodea, and five genera and families associated to the class Hypotrichia, as well as the significant decline of Peniculia.

### Ciliates functional traits and lakes ecosystem functioning

**Figure 5:**
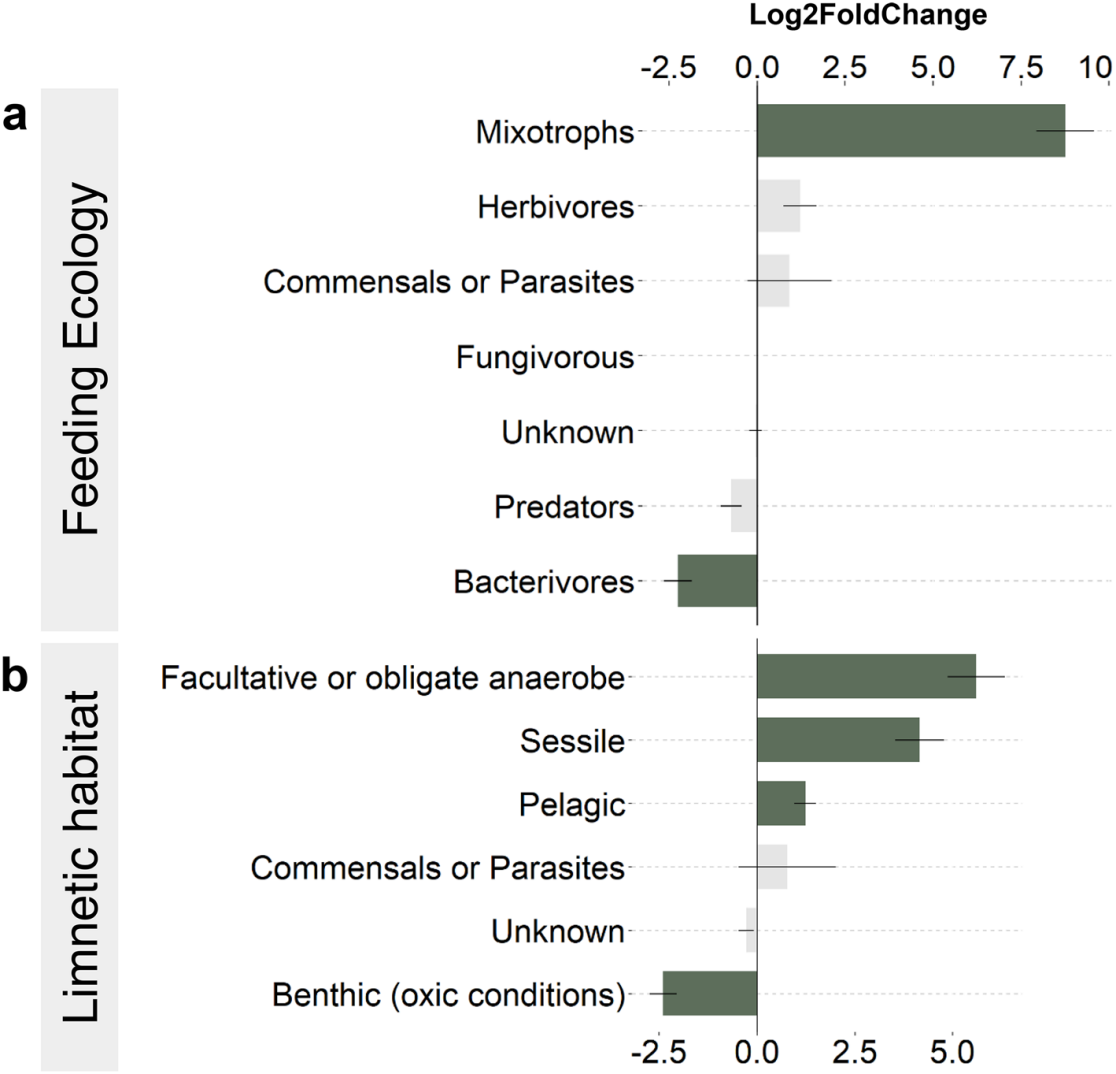
Amplitude of change in the functional groups between the recent and past samples according to (**a**) the feeding ecology and (**b**) limnetic habitat preferences. Magnitude of change is expressed in log2 fold change, as estimated by the DESeq2 analysis (n = 48 lakes). Dark green bars represent groups for which the change was found significant according to the two-sided Wald test corrected with the Benjamini and Hochberg method (p-value < 0.05), while the light grey bars represent groups for which the change was not significant. Horizontal lines show the standard error.

The DESeq2 indicated a significant increase of the facultative or strict anaerobe benthic ciliates while the benthic ciliates that require oxygen significantly declined in the recent period (Fig. 5).

This increase in facultative or anaerobe benthic ciliates was mostly influenced by the significant increase in the abundance of the genus Metopus (order: Armophorida, class: Armophorea) (Supplemental Fig. S3). Moreover, there was a significant increase in sessile ciliates (i.e. attached to a substrate), as well as a significant increase in the pelagic ciliates. In addition, the same analysis applied on the foraging trait categories indicated a significant increase in mixotrophs, while bacterivores significantly declined in recent samples (Fig. 5).

## Discussion

The present study represents the first paleolimnological reconstruction of ciliate communities. Over the last decades, lakes have been exposed to environmental changes (anthropogenic stressors and natural changes) with important implications on the biological communities. By using DNA-based methods, recent paleolimnological studies have provided new insights on the long-term responses of microbial assemblages^15^. Previous paleolimnological investigations of overall microeukaryotes communities reported a strong response of the ciliates to nutrients inputs^13,31^. However, long-term changes in ciliates communities’ structure and functional ecology had not been investigated yet. Through the use of primers that specifically target ciliates, our study brings an innovative perspective. The results highlight changes in heterotrophic and mixotrophic communities that were not previously revealed in the analysis of the overall microeukaryote community (i.e. using generalist primers). Overall, our study demonstrates the potential of using these protists as indicators of environmental change in paleoenvironmental reconstructions. Analyses of the ciliate communities indicate an overall decline in the β-diversity in recent times following the same trend as the overall microeukaryotes diversity^18^. Interestingly, the ciliates did not undergo a large turnover. Instead, our results indicate a spatial homogenization of the diversity with a reorganisation of the community structure (i.e. switch in dominance) (Figs. 2 and 3).

Past ciliate communities were initially heterogeneous at the geographical scale of our study, with some similarities in the community composition for lakes belonging to the same geological regions (Fig. S2). For instance, lakes located on late Paleozoic igneous and metamorphic bedrocks (i.e. Massif Central and Vosges) were characterized by past communities dominated by Colpodea. Bedrock geology can strongly influence the physico-chemical properties of lakes, thereby having subsequent consequences on the natural spatial distribution of communities^32–34^. The heterogeneity of past communities was thus likely driven by historical environmental conditions, and stands in contrast with the more homogeneous composition of the recent ciliate communities across all lakes.

Biotic homogenization of communities is a well documented phenomenon. Previously observed in both terrestrial and aquatic ecosystems, biotic homogenization is strongly influenced by the decline in environmental heterogeneity and availability of diverse ecological niches^35–37^. In aquatic ecosystems, change in climate, productivity, and anthropogenic alteration of the watershed are the most prevalent causes of biotic homogenization^38^. Several of our studied lakes are exposed to similar stressors which includes nutrient enrichment, agricultural and urban development of the watershed and climate change^39–42^. As such, these factors likely acted as deterministic filters selecting for a more homogeneous group of species dominated by generalist ciliates that display more flexible life strategies.

The recent increase in the mixotrophic ciliates is a marked modification of the ciliate communities in response to new environmental conditions^43^. Mixotrophic ciliates have been found to be abundant in the epilimnion of stratified oligotrophic lakes^44^, as well as at the oxic-anoxic boundary of eutrophic freshwater ponds^45^. Moreover, mixotrophic organisms have been found to thrive during transition phases between autotrophy-dominated and heterotrophy-dominated systems^46,47^. As such, they can easily adapt when exposed to extreme events or highly-variable environmental conditions^46,47^, which have been more frequent over the last decades^48^. In our studied lakes, the genera *Limnostrombidium* spp. and *Uroleptus* spp. (Spirotrichea) are the dominant mixotrophic ciliates. Both display mixotrophic life strategies whereby they harbor picoplanktonic species as algal endosymbionts^43,49^. As such, changes in autotrophic picoplankton dynamic and structure previously recorded in some of our studied lakes^16^, might have provided them with a competitive advantage. Empirical studies are still needed in order to better understand the underlying mechanisms benefiting mixotrophic life strategies. Nonetheless, recent increase in the mixotrophic ciliates suggests that lakes might have undergone important trophodynamic changes as mixotrophy is becoming an increasingly important pathway in aquatic food webs. These results also support the importance of integrating the mixotrophic component when studying and modelling aquatic food webs^50^.

The spatial homogenization of the ciliate communities is also marked by the replacement of three clusters by one homogeneous community dominated by Haptoria across all lakes. Haptoria is a widespread subclass of ciliates that typically dominates planktonic communities of both eutrophic and oligotrophic freshwater environments^51–53^. As micropredators, they occupy an intermediate position within the microbial loop^54^, where they are strongly exposed to both bottom-up and top-down forces^55^. These complex interactions are still poorly understood. As such, it remains difficult to achieve a comprehensive picture of the processes that structure these heterotrophic micropredator communities. Nonetheless, given their pivotal role as grazers of other ciliates, algae and bacteria and as source of food for the metazooplankton, such changes in predatory ciliates abundance can have important consequences on the structure and functioning of aquatic food webs^55^.

Changes in several other functional groups of ciliate provide additional evidence of recent modification of the aquatic food web structure and habitat. For instance, the significant increase in pelagic ciliates support that lakes might have been exposed to longer and stronger periods of stratification in recent time. Indeed, these ciliates tends to be found in higher abundance in the epilimnium of stratified lakes^27,52,56,57^. Furthermore, the significant increase in periphytic species, such as the sessile or sedentary forms Peritrichia and Suctoria, is consistent with more frequent pelagic blooms or increased macrophytes growth under higher nutrient and warmer conditions^58,59^.

In parallel to the changes observed in the pelagic ciliates, their benthic counterparts were also largely modified, indicating that the benthic environment has also been impacted by recent environmental changes. The significant increase in the facultative or obligate anaerobic ciliate, such as *Metopus*, suggests that ciliate communities have been directly influenced by the widespread deoxygenation of temperate lakes^5^ (Fig. 5 and Supplemental Fig. S3). The significant decline in the benthic and hypolimnitic ciliates associated with well-oxygenated conditions further support that the habitability of the sediment–water interface has been declining for this particular group of ciliates. The depletion of oxygen concentrations in the profundal zone of freshwater lakes is a well recorded global phenomenon that can have a pervasive impact on the overall ecosystem functioning^60^. These changes have been associated with stronger and longer thermal stratification, as well as a loss of water clarity, in part due to the increases in pelagic production^5^. Supporting this hypothesis, increase in the strict anaerobe bacteriophage *Metopus* was recorded in lakes that are currently categorized as eutrophic or have experienced unprecedented episodes of eutrophication or cyanobacterial bloom over the last decades, and subsequent periods of deep water hypoxia^61–63^.

Although the homogenisation of the beta diversity is a global pattern that we observed, some lakes were more particularly affected in terms of magnitude of change. A stronger restructuration of the ciliate communities was observed in the low elevation lakes, thereby demonstrating that environmental changes in lowland lakes impacted several trophic levels, including non-photosynthetic protist communities. The geographical variation in the amplitude of changes in diversity and community turnover of microorganisms associated with an elevation gradient have been previously demonstrated in terrestrial^64^ and aquatic ecosystems^65^. These patterns are explained by a more pronounced human footprint in lowlands^64,66^, which includes among others, the nutrient-enrichment of freshwater ecosystems^6^. Supporting this trend, the present day trophic status of our studied lakes was significantly higher for lowland lakes than for high elevation lakes (Supplemental Fig. S4). Altogether, our results thus suggest that human-induced nutrient increase influenced the observed changes in the ciliate community diversity of lowland lakes.

Overall, our results provide evidence that the ciliate communities are strongly responding to various environmental factors which includes widespread deoxygenation of deep waters, changes in thermal stratification and nutrient-enrichment. Playing a key role in the metabolic pathways of aquatic ecosystems^53,67^, they can provide valuable insight into the functional ecology of lakes. Although more empirical studies are needed in order to better identify the underlying mechanisms involved, the strong response of ciliates recorded in the sedimentary archives suggests important changes in the main pathways for the transfer of energy within the microbial food webs^68^.

Altogether, the diagnosis of the changes in the ciliate communities across the 48 studied lakes supports the use of ciliates as indicators of environmental changes^69^. Working with ancient DNA and metabarcoding of protists are quite challenging, as such, several aspects need to be taken into consideration for future studies (as summarized in Methods section of the Supplemental Material S1). Nonetheless, the present study as well as several previous investigations^27,67,70^ have shown the great success of using such approaches to assess environmental changes in aquatic ecosystems. Their integration to environmental assessment using high-throughput sequencing and metabarcoding technologies is thus promising, as they provide a more holistic view of the response of aquatic ecosystems to environmental changes. This is even more relevant as the science is moving toward ecosystem-wide food web modelling^20,67,71^, and protists, as key players of the microbial food-web, serve an important function of recycling carbon and energy in lakes.

## Methods

### Study sites and sediment core collection

A total of 48 lakes were used in this study (Fig. 1, Supplemental Table S3). The studied lakes were selected because they were located along a large elevation gradient and displayed various physico-chemical characteristics (Supplemental Table S3). For all 48 lakes, the sediment cores were retrieved between 2010 and 2016 (cf. Keck et al.^18^ for more detailed information on each lake) from the deepest part of the basin using a UWITEC gravity corer. Prior to subsampling, the core was air protected by a double layer of plastic wrap and stored in the dark at 4 °C.

### Methodological considerations and sample selection for sedaDNA analysis

A top–bottom technique was used to provide a simplified assessment of the amplitude of change in the ciliate community diversity and composition. Briefly, a top sample corresponding to recent time and a bottom sample corresponding to the past were isolated from the cores.

The level of DNA preservation in the sediment (from one lake to another or when aging in sediment) is a sensitive point to be taken into account for paleo-reconstruction studies, and for sample selection. Aquatic sediments are, a priori, suitable environments for DNA preservation through the binding to mineral and organic particles, and the absence of oxygen and UV radiation^72,73^. Nonetheless, several processes can alter DNA sequences in marine^74^ and freshwater sediments^15^. It was thus important to consider whether the differences observed between top and bottom strata could be induced by diagenetic processes responsible for the modification of DNA signal over time. The potential distortions to lake sediment DNA records due to taphonomic processes (production, transfer, preservation of DNA) that affect DNA in sediments are not fully known. However, we know that (1) the DNA signal is proven to be reliable for several centuries in lake sediments and the signal can be preserved for several millennia if the preservation conditions are very good^75^; (2) the first few years after deposition are critical for DNA preservation due to the biological activity at the sediment–water interface and the physical and chemical changes that occur in the uppermost sediment layers^12^. To overcome this issue, the top strata were sampled a few centimeters down the surface of the core (~ year 2000 C.E.) in order to minimize biases associated with early diagenesis processes and active benthic ciliates (more details about the selection of the samples and methodological considerations can be found in the online Supplemental Material S1). The depth of the bottom samples was chosen for each core in order to correspond to the pre-“Great Acceleration” period^2,3^ (i.e. nineteenth century) which was determined using a combination of several approaches (i.e. X-ray fluorescence, radiocarbon and radionuclides ^210^Pb and ^137^Cs; cf. the Supplementary Material Methods S1 and Table S4). In order to ensure that each sample covered at least 10–15 years, the thickness of the sediment samples was individually adjusted (cf. the Supplementary Material Methods S1 and Table S4). Sub-sampling for DNA analysis was conducted in a clean and controlled environment using strict laboratory protocols to avoid contamination by modern DNA^15,18^.

### Molecular analysis

Two DNA extractions were performed on 0.5 g of wet sediment for each sample using the NucleoSpin® soil kit, according to the manufacturer instructions (Macherey–Nagel, Düren, Germany). The same DNA extracts as in Keck et al.^18^ were used. Refer to the Method section of the Supplementary Material S1 for more details regarding the laboratory protocol and conditions applied for the DNA extraction. A nested-PCR targeting the V4 region of the 18S rRNA gene was used to do the inventory of the ciliate community. In the first step, a set of primers was used to target a specific DNA region for ciliates of 800 bp CS322F (5ʹ-GATGGTAGTGTATTGGAC-3ʹ) and 1147R (5ʹ-GACGGTATCTRATCGTCTTT-3ʹ)^76,77^. The first PCR was performed in a total volume of 25 µL containing 1 µL of DNA extract, 2.5 µL of 10X NH4 reaction buffer, 2 µL of 50 mM of MgCl2, 0.5 µL of 100 mM dNTP, 1.25 µL of each primer at 10 pmol/µL, 2 µL of 10 mg/mL BSA and 0.1 µL of 5 Ci BioTaq (Bioline). The amplification cycle included an initial denaturation at 95 °C for 10 min followed by 20 cycles of 15 s at 94 °C, 15 s at 57 °C and 30 s at 72 °C. The amplicons were then subjected to a final 10 min extension at 72 °C. The second PCR was then applied on the products of the first PCR using general eukaryotic primers NSF573 (5′-CGCGGTAATTCCAGCTCCA-3′) and NSR951 (5′-TTGGYRAATGCTTTCGC-3′)^78^, amplifying DNA fragment of about 378 bp. Molecular tails were added to the forward primer (5′-CTTTCCCTACACGACGCTCTTCCGATCT-3′) and to the reverse primer (5′-GGAGTTCAGACGTGTGCTCTTCCGATCT-3′). The second PCR was performed in a total volume of 25 µL containing 0.8 µL of DNA from the first PCR, 2 µL of 10X NH_4_ reaction buffer, 1.6 µL of 50 mM of MgCl_2_, 0.4 µL of 100 mM dNTP, 1 µL of each primer with molecular tails at 10 pmol/µL, 1.6 µL of 10 mg/mL BSA and 0.06 µL of 5 Ci BioTaq (Bioline). The amplification cycle included an initial denaturation at 95 °C for 2 min followed by 20 cycles of 30 s at 94 °C, 30 s at 57 °C and 45 s at 72 °C. The amplicons were then subjected to a final 10 min extension at 72 °C. The nested-PCR protocol was applied on each DNA extraction separately, the full volume of the final products resulting from the two DNA extracts of the same sample were then pooled and sent to GeT-PlaGe (Plateforme Génomique 31326 CASTANET-TOLOSAN Cedex) for amplicon purification, library preparation and paired-end (2 × 250 bp) sequencing on a MiSeq Illumina instrument (San Diego, CA, USA).

The reads were demultiplexed and R1/R2 reads assembled into contigs by the sequencing platform who provided one fastq files per sample. The high-throughput sequencing data were then cleaned in Mothur 1.45.1^79^. Filtering steps were used to conserve DNA sequences of 350 ± 50 bp in length, with no ambiguities (N = 0), 10 or less homopolymer (max homopolymer = 10) and no mismatch was allowed in the primer sequence. The data was dereplicated in order to work with Individual Sequence Unit (ISU). ISUs were then aligned using an aligned version of the Silva 18S database restrained to the V4 region and ISUs that were not fully aligned to the Silva 18S barcode were removed. The detection and removal of chimera was done using Vsearch as implemented in Mothur with default parameters. The taxonomic assignment of the ISU was done using a curated version of the Protist Ribosomal Reference database PR2^80^ “pr2_version-4.12.0_18S_cil_cur” (available on Zenodo repository system: https://doi.org/10.5281/zenodo.5163167) and using the command classify.seqs() and the method wang with a confidence score threshold of 75% and 100 iterations. Following this first taxonomic assignment, the ISU represented with only one read or that were identified as “unknown” or “Eukaryota_unclassified” were removed. The ISUs were then clustered into molecular Operational Taxonomic Unit (OTU) using the furthest neighbour algorithm with a similarity threshold of 97%, as previously suggested by Stoeck et al.^81^. Finally, the command classify.otu() was used to taxonomically assign the OTUs based on the first taxonomic assignment of the ISUs with a confidence threshold of 80%. The OTUs that did not belong to the Phylum Ciliophora were removed. The taxonomic affiliations were checked and harmonized manually using the classification from Gao et al.^82^. In order to study changes in the functional groups, the ecological preferences (i.e. preferred limnetic habitat or foraging traits; cf. Table 1) were indexed for the OTUs for which the taxonomic affiliation was fine enough (at least assigned to the family rank); otherwise, the category “Unknown” was given. The association of OTUs to their functional traits was done through an exhaustive literature review. The foraging traits categories created were inspired from a combination of several previously published categories based on the feeding ecology of ciliates^22,53,69,83^. Table used with the information about the functional traits is available on open access (http://doi.org/10.5281/zenodo.5534333).

**Table 1:** Description of the ciliates functional traits categories.

Categories associated with the limnetic habitat	Benthic (oxic conditions)	Ciliates that can be found in the littoral zone or well-oxygenated bottoms (some of these benthic ciliates can migrated to the anoxic–oxic layer of the pelagic zone when the bottom of the lake become anoxic during stratification periods)
	Commensals or Parasites	Freshwater ciliates that are either endocommensals of bivalves or ectocommensals of fish, or parasites of fish
	Facultative or obligate anaerobe	Facultative or obligate anaerobe living in the benthic environment but also includes some taxa able to live in anaerobic deep waters
	Pelagic	Euplanktonic ciliates
	Sessile	Ciliates for which their life cycle includes a stage attached to a substrate (usually stalked ciliates)
Categories associated with foraging strategies	Herbivorous	Algivores
	Bacterivores	Ciliates that exclusively feed on bacteria, these ciliates are usually associated with the benthic environment or found in the metalimnion of highly productive lakes
	Predators	Regroup: (1) Ciliates that feed on other ciliates or even small metazoans; (2) Omnivorous ciliates feeding on algae, bacteria and other small ciliates
	Commensals and parasites	Regroup parasites, bacterivores and histophage ciliates; commensals and parasitic ciliates were kept separated, as they are more likely to be directly influenced by the presence/absence of their hosts rather than influenced by changes in the biotic and abiotic factors of the surrounding environment
	Fungivorous	This category contains only one species found in our samples *Pseudoplatyophrya nana*
	Mixotrophs	Phagotrophic ciliates that harbor algal endosymbionts or sequester plastids from their algal prey; mixotrophic ciliates thus tend to be also algivores

### Statistical analysis

Analyses were done using the R software version 3.11^84^ using the *vegan* package^85^, the *rpart* package^86^ and DESeq2 package^30^.

The normality and homogeneity of variance of the environmental variables were tested using a Shapiro–Wilk test of normality^87^ and a Fligner-Killeen’s test^88^, respectively. If the variables were not normally distributed even after transformation non-parametric test were used. A Spearman correlation analysis was applied to study the relationship between the environmental variables. To assess the relationship between lake topology and trophic status the Kruskal test was used; whenever the Kruskal test^89^ was significant, in order to assess which categories of trophic status were significantly different, a Wilcoxon rank sum test as Post Hoc test was applied using the False Discovery Rate approach by Benjamini and Hochberg^90^ to adjust the p value for multiple testing (Supplemental Fig. S4).

In order to harmonise data and allow comparison between samples, reads were transformed into relative abundance. Changes in the β-diversity of the ciliates at the community level between the past and recent samples were investigated using a Bray–Curtis dissimilarity matrix that was built based on the relative abundance data at the subclass level. Results were visualized on a NMDS (Non-metric Multidimensional Scaling analysis). To evaluate the change in dispersion and thus in diversity between the past and recent samples, the distances between the samples and the geometric median for each group (“recent” and “past”) were calculated. The difference between the median of each group was then tested using the Wilcoxon rank sum test in order to evaluate the overall displacement of the recent and past samples. A hierarchical cluster analysis on Bray–Curtis distances using unweighted pair group method with arithmetic mean (UPGMA) was used to identify if a clear separation could be observed between and within recent and past samples. A SIMPER analysis (SIMilarity PERcentage)^91^ was performed on the relative abundance data to calculate the contribution of each subclass to the overall Bray–Curtis dissimilarity between the recent and past samples. The most abundant species can have a high contribution even when they do not differ among groups, as they tend to display the highest variance^85^, as such, the proportion in SIMPER contribution, in average change between recent and past samples, as well as the total number of reads were compared to each other.

In order to evaluate the relative importance of known physical characteristic of the lakes (i.e. continuous variables: elevation, maximum depth, surface area of the lake, surface area of the watershed) to the amplitude of changes in the β-diversity, a univariate regression tree analysis was applied on the Bray–Curtis dissimilarity between the recent and past sample of each lake. For categorical data (i.e. Trophic Status), an analysis of variance (ANOVA) was used to compare the Bray–Curtis dissimilarity coefficients between categories.

The difference in abundance between the recent and past samples was also evaluated for each functional group and at the Genus level using the DESeq2 framework applied on the raw count data^30^. The results were expressed as “log2foldchange” and provided an indication of the intensity of the changes between the recent and past samples.

For the creation of the map, the software QGIS^92^ was used, for all other figures, we used the package *ggplot2*^93^ and the color palette from the package *scico*^94^ to create scientifically derived color-maps accessible for people with colour-vision deficiencies^95^.

## Supplementary Information

Supplementary Information.

## Data Availability

Raw metabarcoding data are available at https://doi.org/10.5281/zenodo.5163167. Taxonomical and functional trait affiliation used are available at http://doi.org/10.5281/zenodo.5534333.
