# Supplementary material for: Paleoreconstructions of ciliate communities reveal long-term ecological changes in temperate lakes
Source: Sci Rep. 2022 May 12;12:7899. doi: 10.1038/s41598-022-12041-7 (PMC9098483; doi:10.1038/s41598-022-12041-7)
Supplement: Supplementary file 1 — Supplementary Information. [file 41598_2022_12041_MOESM1_ESM.docx]

**Supplementary Material for the article**

Paleoreconstructions of ciliate communities reveal long-term ecological changes in temperate lakes

Barouillet Cécilia*^1,2^, Valentin Vasselon^1,2,3^, François Keck^1,2^, Laurent Millet^4^, David Etienne^5,2^, Didier Galop^6,7^, Damien Rius^4^ & Isabelle Domaizon*^1,2^

^1^ INRAE, Université Savoie Mont Blanc, CARRTEL, 74200 Thonon-les-Bains, France.

^2^ Pole R&D ECLA, CARRTEL, 74200 Thonon-les-Bains, France.

^3^ OFB, Site INRAE UMR CARRTEL,74200 Thonon-les-Bains, France

^4^ CNRS, Chrono Environnement, 25000 Besançon, France.

^5^ Université Savoie Mont Blanc, INRAE, CARRTEL, 73370 Le Bourget du Lac, France.

^6^ GEODE UMR 5602 CNRS, Université de Toulouse, 31058 Toulouse, France.

^7^ Labex DRIIHM, OHM Pyrénées, CNRS/INEE, Toulouse, France.

**Methods**

Sediment dating and selection of the top and bottom samples:

We combined chronological information from previous studies (when available) with chronostratigraphic markers such as atmospheric pollutant (lead), short-lived radionuclides (^137^Cs/^210^Pb), and radiocarbon dating. Geochemical analyses, including lead and other trace elements, were performed by X-ray fluorescence (AVAATECH XrF Core Scanner, Edytem Laboratory) at a 5 mm sampling step. Radionuclides ^137^Cs and ^210^Pb were measured by gamma spectrometry (Chrono-Environnement Laboratory). Radiocarbon measurements were performed using accelerator mass spectrometry in Poznan Radiocarbon Laboratory, Poland. The main challenge in dating lacustrine sediments from the past two centuries is to estimate ages at the onset to the mid-19th century. Stratigraphic profiles of stable lead (Pb) were used to synchronize the lake sediment records with historical lead atmospheric deposition. More specifically, the study area was more particularly characterized by the increase in Pb fallout from the mid-1800s to the beginning of the 20th century following the industrial revolution and by the decrease in Pb deposition at the end of the 1970s following the decline in the use of lead additives in European countries. Eventually, chronostratigraphic markers such as lead can be differentially recorded in lakes, depending on the local human impact history (mining) sedimentation rate, and sediment geochemical properties. For all these reasons, it can be necessary to combine different methods, or to choose a specific one according to lake type.

Supplementary Table S4 summarizes how the depth and thickness of top/bottom sediment layers were set.

The thickness was defined as follows:

1. Using the depth of stable lead decrease from XrF core logging alone.
2. Same as 1 with addition of the depth of 1986 and/or 1963 ^137^Cs atmospheric fallout (Chernobyl accident and maximum of atmospheric nuclear weapon tests, respectively). In this case, the end of the 1970s stable lead peak is constrained between the two ^137^Cs peaks.
3. Using radionuclides (137Cs/210Pb) based age-depth model. The difference with 2 is that ^210^Pb enables to calculate an age for each analysed sediment samples over the last 150 yrs.
4. Using sedimentation rate calculated from ^14^C ages.
5. For high-elevation lakes with low sedimentation rates and without chronostratigraphic marker visible along the upper part of the core, the thickness was set to 1.5 to 2 cm.

For bottom samples, depths and thickness were defined as follows:

1. Using the depth of the onset of stable lead increase from XrF core logging alone.
2. As with 1, with addition of radionuclides based (137Cs/210Pb) age-depth model
3. Using sedimentation rate calculated from 14C ages.
4. For two Pyrenean high-elevation lakes with no chronological information, we had to set the depth of thickness of the bottom sediment layers according to the mean sedimentation rate in other lakes of this sub-region in the dataset, that were more precisely dated.

In any case, the selected bottom sediments are anterior in age to the Great Acceleration.

Precautions for sample handling and other methodological considerations:

At every steps of the analysis of paleoenvironmental DNA, strict laboratory protocols and various methodological precautions were taken to achieve robust results and ensure the validity of our molecular data:

- Sterile disposable materials (labware, gloves, etc.) were used for all lab procedures; during sediment core subsampling, sediment slices were taken using sterilised metal plates.
- Separate stations were organized for subsampling, DNA extractions and PCR amplifications. To prevent contamination with modern DNA, the extraction of DNA from sediment is carried out in specific rooms dedicated to rare DNA, and PCR are prepared under designated working stations. These laboratories are physically separated from other molecular biology laboratories.
- Negative controls are included at 3 steps of the procedure: during subsampling (open tubes containing pure water), extraction (treatment of pure water) and PCR preparation (blank PCR tube). All blanks were found negative for the amplification of 18S rRNA markers; consequently, the blanks were not included in the sequencing library.
- Although the set of ciliate-specific primers used (CS322F and 1147R) are known to be highly specific and resolutive, the amplification of a long DNA fragment (i.e. 800 bp) is not optimal when working on ancient DNA. Future studies might thus consider a different set of ciliate-specific primers which would target a shorter region more suitable for ancient-DNA studies^1^. For the second PCR, we selected short barcodes adapted to the work on sedimentary DNA. The choice of the barcode region was previously explained in Capo et al^2^ based on verifications of the coverage of primers for microeukaryotic diversity, the ability to amplify the DNA with these primers, and the quality of taxonomic assignment obtained, the probes 960F and NSR1438 were selected.
- To evaluate the potential influence of co-extracted inhibitors present in sedimentary DNA extracts (that may reduce the efficiency of downstream PCR), we assessed the inhibition level using quantitative PCR assays. The approach applied is based on the assumption that inhibitors are diluted out when a log-linear relationship is achieved between Cq and the dilution factor^3^. No inhibition effect was found.
- We performed a duplicate extraction for each strata and verified the similarity of DNA results obtained for the replicates of a given strata (see Keck et al. Supplemental Material).
- In compacted lake sediments, vertical advection of pore water is minimal, and multivalent metals and organic compounds (pigments, organic molecules with more than 15 carbon atoms) are immobilized in the sediment matrix^4^. Large organic molecules such as DNA are likely to adhere to solid-phase sediments (particles, particulate organic matter) or are locked in dead cells or ancient dormant resting cells. Therefore, leaching of DNA is unlikely to occur in lake sediment and lake sedimentary DNA is assumed to give an accurate temporal reconstruction of the biological community succession^4,5^. The level of DNA preservation in the sediment (from one lake to another or when aging in sediment) is a sensitive point to be taken into account for paleo-reconstruction studies. Given the mechanisms of DNA protection by binding to mineral and organic particles and due to the absence of oxygen and UV radiation, aquatic sediments are, a priori, suitable environments for DNA preservation^6,7^. However, several processes can alter DNA sequences in marine^8^ and freshwater sediments^9^. It was thus important to consider whether the differences observed between top and bottom strata could be induced by diagenetic processes responsible for the modification of DNA signal over time. Though shotgun sequencing allows to differentiate ancient DNA that has been damaged (typical damage patterns of ancient DNA marked by increase in T and A at the ends of DNA fragments), the limited number of samples that can be treated in parallel and the associated cost per sample still limit routine application when a large number of samples are to be treated (as here with 96 × 2 samples). The potential distortions to lake sediment DNA records due to taphonomic processes (production, transfer, preservation of DNA) that affect DNA in sediments are not fully known; we know however that:
  - At sites with favourable DNA preservation conditions like in lake sediments, the DNA signal is proven to be reliable for several centuries. The signal can be preserved for several millennia if the preservation conditions are very good^10^.
  - The first few years after deposition are critical for DNA preservation due to the biological activity at the sediment interface and the physical and chemical changes that occur in the uppermost sediment layers^2^. Consequently, we chose to avoid the very recent deposits (for the sampling of modern periods) to overcome this issue; the top samples were sampled a few centimeters below the sediment surface (~year 2000).
- Different levels of taxonomy were considered throughout the data analysis in order to circumvent the potential risk associated with the use of OTUs (artifact increase of OTUs number, or loss of OTUs due to degradation/fragmentation). The choice of thresholds for the delineation of OTUs is critical, with potential risk of inflation of rare OTUs or, inversely, of lumping together OTUs with different distribution patterns. Universal thresholds also do not consider differences in substitution rates among lineages and may therefore not capture equivalent units of diversity.
- In the present study, the top strata were sampled a few centimeters down the surface of the core in order to minimize bias associated with early diagenesis processes and living taxa. However, the presence of 59 OTUs specific to the top strata could either be related to the changes in environmental conditions in recent time or be an artifact associated with taxa living in the subsurface layers of the sediment. Importantly, the number of OTUs specific to the top strata remains low (~2% of the total OTUs) and the increase of *Metopus* genera, a ciliate associated with anoxic conditions, in the top strata supports that the observed changes likely track changes in the community composition over time. Nonetheless, a better understanding of post-depositional survival and activity of microorganisms is still required, and becomes even more important when using the top-bottom paleolimnological technique in order to insure the authenticity of the sed-DNA molecular signal as an archive of past environmental conditions^11^.
- Molecular studies of protist communities present some challenges related to the lack of a robust reference database for ciliates^12^. More robust reference database would improve functional traits affiliation which would allow for a better understanding of the functional ecology of ciliates and associated studied of lake ecosystem functioning.
- Both intracellular DNA and extracellular DNA were extracted using the NucleoSpin® soil kit. Recovering the DNA signal from both fractions allows obtaining a better representation of overall biodiversity. Although the DNA signal in marine sedimentary archives is mostly contained in the extracellular DNA fraction^13^, the comparison of metabarcoding results obtained for extracellular DNA and intracellular DNA (lake sediments) showed that extracting only extracellular DNA tends to results in lower yield and lower diversity for micro-eukaryotic taxa^11^; this is therefore recommended to work on total DNA.
- One hypothesis regarding the DNA preservation during burying into sediments is that extracellular DNA could be less protected than intracellular DNA (physically protected within resistant cells). Cyst probably influence the sinking through the water column, however no study have clearly demonstrated that the DNA of taxa forming cyst is better preserved in sediments. Capo et al.^14^ who studied the effect of early diagenesis on the preservation of microeukaryotic DNA in lake sediments, found no link between the relative abundance of DNA reads and the putative level of cellular DNA protection (capacity of taxa to form resistant cells, e.g. spores, cysts) for micro-eukaryotic taxa. Additionally, even though intracellular DNA is protected against external factors when inside protective resting stages such as cysts or spores, DNA molecules can nonetheless be degraded by intracellular nucleases upon cell death^11^. In contrast, extracellular DNA that has been released into the environment upon cell lysis can be quickly absorbed by clay minerals, which significantly promotes its preservation by making DNA protected from chemical/physical degradation processes^15^ and less accessible as a food source for indigenous sediment bacteria^13^.
- A potential challenge related to the molecular approach is the overrepresentation of some species that contains numerous copies of the targeted gene per cell^16^. A few studies already compared environmental DNA metabarcoding with microscopic analysis of ciliates communities^17–19^. All concluded that, by integrating information from rare species and resting stages, the molecular approach consistently detect a higher diversity, also allowing to integrate a broader view of the ciliate community. For the few ciliates for which we have information about their number of copies per cell, there was no clear relationship between the number of DNA reads and the number of copies of gene per cell (Supplemental Fig. S5). Additionally, the ecological interpretations based on the relative comparison of the top and bottom samples from the same lake (which have exposed to similar preservation conditions) would unlikely be affected by this artifact. Moreover, analytical methods such as the DeSeq2 analysis applied on our comparative top-bottom approach reduce interpretation errors related to these potential biases^20^.

1. Pitsch, G. *et al.* Seasonality of Planktonic Freshwater Ciliates: Are Analyses Based on V9 Regions of the 18S rRNA Gene Correlated With Morphospecies Counts? *Front. Microbiol.* **10**, 248 (2019).

2. Capo, E. *et al.* Tracking a century of changes in microbial eukaryotic diversity in lakes driven by nutrient enrichment and climate warming: Long-term dynamics of microbial eukaryotes. *Environ Microbiol* **19**, 2873–2892 (2017).

3. Lloyd, K. G., MacGregor, B. J. & Teske, A. Quantitative PCR methods for RNA and DNA in marine sediments: maximizing yield while overcoming inhibition. *FEMS Microbiology Ecology* **72**, 143–151 (2010).

4. Anderson-Carpenter, L. L. *et al.* Ancient DNA from lake sediments: Bridging the gap between paleoecology and genetics. *BMC Evol Biol* **11**, 30 (2011).

5. Ficetola, G. F. *et al.* DNA from lake sediments reveals long-term ecosystem changes after a biological invasion. *Sci. Adv.* **4**, eaar4292 (2018).

6. Ogram, Andrew., Sayler, G. S., Gustin, Denise. & Lewis, R. J. DNA adsorption to soils and sediments. *Environ. Sci. Technol.* **22**, 982–984 (1988).

7. Parducci, L. *et al.* Shotgun Environmental DNA, Pollen, and Macrofossil Analysis of Lateglacial Lake Sediments From Southern Sweden. *Front. Ecol. Evol.* **7**, 189 (2019).

8. Pedersen, M. W. *et al.* Ancient and modern environmental DNA. *Phil. Trans. R. Soc. B* **370**, 20130383 (2015).

9. Domaizon, I., Winegardner, A., Capo, E., Gauthier, J. & Gregory-Eaves, I. DNA-based methods in paleolimnology: new opportunities for investigating long-term dynamics of lacustrine biodiversity. *J Paleolimnol* **58**, 1–21 (2017).

10. Epp, L. S. A global perspective for biodiversity history with ancient environmental DNA. *Mol Ecol* **28**, 2456–2458 (2019).

11. Capo, E. *et al.* Lake Sedimentary DNA Research on Past Terrestrial and Aquatic Biodiversity: Overview and Recommendations. *Quaternary* **4**, 6 (2021).

12. Sagova-Mareckova, M. *et al.* Expanding ecological assessment by integrating microorganisms into routine freshwater biomonitoring. *Water Research* **191**, 116767 (2021).

13. Corinaldesi, C., Danovaro, R. & Dell’Anno, A. Simultaneous Recovery of Extracellular and Intracellular DNA Suitable for Molecular Studies from Marine Sediments. *Appl Environ Microbiol* **71**, 46–50 (2005).

14. Capo, E., Domaizon, I., Maier, D., Debroas, D. & Bigler, C. To what extent is the DNA of microbial eukaryotes modified during burying into lake sediments? A repeat-coring approach on annually laminated sediments. *J Paleolimnol* **58**, 479–495 (2017).

15. Kanbar, H. J., Olajos, F., Englund, G. & Holmboe, M. Geochemical identification of potential DNA-hotspots and DNA-infrared fingerprints in lake sediments. *Applied Geochemistry* **122**, 104728 (2020).

16. Zhao, F., Filker, S., Stoeck, T. & Xu, K. Ciliate diversity and distribution patterns in the sediments of a seamount and adjacent abyssal plains in the tropical Western Pacific Ocean. *BMC Microbiol* **17**, 192 (2017).

17. Boscaro, V. *et al.* Strengths and Biases of High-Throughput Sequencing Data in the Characterization of Freshwater Ciliate Microbiomes. *Microb Ecol* **73**, 865–875 (2017).

18. Stoeck, T. *et al.* A morphogenetic survey on ciliate plankton from a mountain lake pinpoints the necessity of lineage‐specific barcode markers in microbial ecology. *Environ Microbiol* **16**, 430–444 (2014).

19. Kulaš, A. *et al.* Ciliates (Alveolata, Ciliophora) as bioindicators of environmental pressure: A karstic river case. *Ecological Indicators* **124**, 107430 (2021).

20. Love, M. I., Huber, W. & Anders, S. Moderated estimation of fold change and dispersion for RNA-seq data with DESeq2. *Genome Biol* **15**, 550 (2014).

**Table S1:** Summary of the effect of the bioinformatic steps on the DNA reads per sample. Filtering code (1) raw data obtained from the sequencing platform, (2) conserve DNA sequences of 350±50 bp in length, with no ambiguities (N=0), 10 or less homopolymer (max homopolymer=10), (3) conserve DNA sequences with primers (no mismatch was allowed in the primer sequence), (4) ISUs aligned using an aligned version of the Silva 18S database restrained to the V7 region, removal of ISUs that were not fully aligned to the Silva 18S barcode, (5) Removal of Chimera, (6) taxonomic assignment of the ISU, and (7) removal ISU represented with only one read or that were identified as “unknown” or “Eukaryota_unclassified”. The Final column corresponds to the final number of reads obtained after OTU clustering using the furthest neighbor approach with a similarity threshold of 97%.

| **Filtering code** | **1** | **2** | **3** | **4** | **5** | **6** | **7** |  |
| --- | --- | --- | --- | --- | --- | --- | --- | --- |
| **Sample_ID** | **Contigs** | **Trim**  **length_homop_N** | **Trim**  **primer** | **Trim**  **Alignment** | **Trim**  **Chimera** | **Trim**  **tax** | **Trim**  **ISU>1** | **Final** |
| **ABB_B** | 32791 | 32768 | 29622 | 29555 | 29388 | 29381 | 20827 | 20827 |
| **ABB_T** | 31861 | 31835 | 29198 | 29016 | 27994 | 27991 | 20817 | 20817 |
| **AIG_B** | 23743 | 23716 | 21009 | 20872 | 20858 | 20840 | 16319 | 16315 |
| **AIG_T** | 25576 | 25550 | 22907 | 22785 | 22322 | 22308 | 16349 | 16349 |
| **ALA_B** | 18905 | 18894 | 16552 | 16500 | 16472 | 16471 | 11087 | 11087 |
| **ALA_T** | 25684 | 25662 | 23206 | 23149 | 23075 | 23075 | 15149 | 15149 |
| **ARA_B** | 26113 | 26071 | 22740 | 22636 | 22096 | 22095 | 16729 | 16729 |
| **ARA_T** | 30682 | 30647 | 27609 | 27515 | 26455 | 26455 | 18960 | 18960 |
| **AYD_B** | 37525 | 37490 | 34131 | 34048 | 33669 | 33665 | 25618 | 25618 |
| **AYD_T** | 41700 | 41655 | 37913 | 37146 | 34746 | 34731 | 25419 | 25419 |
| **AYE_B** | 24459 | 24447 | 21933 | 21900 | 21406 | 21406 | 14921 | 14921 |
| **AYE_T** | 25970 | 25952 | 23678 | 23619 | 22775 | 22775 | 16077 | 16077 |
| **BAR_B** | 32700 | 32604 | 26234 | 25989 | 25955 | 25911 | 20372 | 20372 |
| **BAR_T** | 20525 | 20515 | 18587 | 18386 | 17796 | 17791 | 12142 | 12142 |
| **BARR_B** | 27241 | 27222 | 24693 | 24628 | 24570 | 24226 | 16646 | 16646 |
| **BARR_T** | 17787 | 17780 | 16188 | 16159 | 16131 | 16131 | 11947 | 11945 |
| **BES_B** | 27130 | 27117 | 24869 | 24831 | 24350 | 24349 | 16453 | 16453 |
| **BES_T** | 17272 | 17254 | 15599 | 15188 | 14646 | 14644 | 9510 | 9510 |
| **BLAC_B** | 44998 | 44964 | 40729 | 40633 | 39364 | 39364 | 28729 | 28729 |
| **BLAC_T** | 30069 | 30030 | 27020 | 26577 | 24945 | 24942 | 17960 | 17960 |
| **BON_B** | 19598 | 19573 | 17741 | 17527 | 17462 | 17412 | 12900 | 12900 |
| **BON_T** | 38813 | 38791 | 35711 | 35593 | 34818 | 34818 | 25822 | 25822 |
| **BOR_B** | 23568 | 23555 | 21570 | 21542 | 21486 | 21485 | 17195 | 17195 |
| **BOR_T** | 20319 | 20301 | 18324 | 18271 | 17992 | 17992 | 14058 | 14058 |
| **BOUR_B** | 26926 | 26912 | 24603 | 24492 | 24486 | 24472 | 20502 | 20502 |
| **BOUR_T** | 44554 | 44529 | 40417 | 39039 | 37663 | 37658 | 28434 | 28434 |
| **CHA_B** | 29127 | 29104 | 26549 | 26498 | 26475 | 26475 | 21528 | 21528 |
| **CHA_T** | 37443 | 37411 | 33999 | 33864 | 32964 | 32962 | 24208 | 24208 |
| **CHE_B** | 33782 | 33756 | 30189 | 30129 | 28904 | 28848 | 19326 | 19324 |
| **CHE_T** | 25315 | 25295 | 22671 | 22617 | 22219 | 22183 | 16748 | 16748 |
| **COR_B** | 38132 | 38111 | 34800 | 34748 | 32022 | 32011 | 23333 | 23333 |
| **COR_T** | 32550 | 32536 | 29913 | 29804 | 28480 | 28480 | 20190 | 20190 |
| **CRE_B** | 24472 | 24462 | 22352 | 22313 | 20099 | 20097 | 13925 | 13925 |
| **CRE_T** | 29874 | 29837 | 27087 | 26171 | 23394 | 23389 | 16842 | 16842 |
| **ESP_B** | 31863 | 31831 | 28945 | 28842 | 28064 | 28064 | 19627 | 19624 |
| **ESP_T** | 32238 | 32219 | 29378 | 29265 | 28458 | 28453 | 19155 | 19155 |
| **ETI_B** | 13157 | 13138 | 11657 | 11173 | 11168 | 10661 | 8785 | 8785 |
| **ETI_T** | 29965 | 29941 | 27232 | 27153 | 26713 | 26712 | 19473 | 19473 |
| **GEN_B** | 35088 | 35062 | 32136 | 31998 | 30073 | 30073 | 22675 | 22673 |
| **GEN_T** | 35280 | 35252 | 31584 | 31353 | 28337 | 28337 | 21815 | 21815 |
| **GER_B** | 36483 | 36440 | 33084 | 33018 | 31926 | 31924 | 24362 | 24362 |
| **GER_T** | 24057 | 24040 | 22016 | 20119 | 18631 | 18631 | 13532 | 13532 |
| **GOD_B** | 28919 | 28812 | 23738 | 23697 | 22845 | 22845 | 16949 | 16949 |
| **GOD_T** | 25504 | 25476 | 22170 | 22148 | 22135 | 22135 | 17107 | 17107 |
| **GOU_B** | 24522 | 24501 | 21746 | 21695 | 21617 | 21599 | 15550 | 15550 |
| **GOU_T** | 28835 | 28822 | 26479 | 26380 | 25551 | 25547 | 18715 | 18715 |
| **GUE_B** | 39150 | 39104 | 32940 | 32805 | 32393 | 32391 | 24929 | 24929 |
| **GUE_T** | 15706 | 15681 | 13783 | 13679 | 12626 | 12626 | 9037 | 9037 |
| **ILA_B** | 21376 | 21356 | 18935 | 18919 | 18785 | 18785 | 13824 | 13824 |
| **ILA_T** | 20210 | 20198 | 18588 | 18506 | 17282 | 17279 | 12525 | 12525 |
| **ISA_B** | 40458 | 40387 | 35698 | 35555 | 35031 | 35030 | 27756 | 27756 |
| **ISA_T** | 23029 | 23013 | 20800 | 20682 | 19834 | 19834 | 14277 | 14277 |
| **LAG_B** | 22377 | 22360 | 20331 | 20289 | 20203 | 20202 | 15328 | 15328 |
| **LAG_T** | 42294 | 42269 | 38125 | 37982 | 35856 | 35856 | 26161 | 26161 |
| **LAM_B** | 42929 | 42879 | 38936 | 38863 | 37615 | 37615 | 26347 | 26347 |
| **LAM_T** | 21474 | 21446 | 19155 | 18925 | 18083 | 18078 | 12484 | 12484 |
| **LAN_B** | 34345 | 34325 | 30921 | 30848 | 29449 | 29449 | 20328 | 20328 |
| **LAN_T** | 24805 | 24783 | 22539 | 22350 | 20576 | 20573 | 15320 | 15320 |
| **LEM_B** | 22926 | 22906 | 20640 | 20613 | 20609 | 20609 | 17155 | 17155 |
| **LEM_T** | 33926 | 33873 | 29554 | 29309 | 28095 | 28093 | 21740 | 21740 |
| **LON_B** | 25589 | 25578 | 23143 | 23009 | 22764 | 22762 | 15399 | 15399 |
| **LON_T** | 26738 | 26724 | 24594 | 24432 | 23227 | 23210 | 16800 | 16800 |
| **MAI_B** | 24442 | 24414 | 21442 | 21389 | 21293 | 21293 | 14537 | 14537 |
| **MAI_T** | 17318 | 17309 | 15519 | 15467 | 15041 | 15038 | 9664 | 9664 |
| **MAR_B** | 48321 | 48278 | 43800 | 43623 | 41680 | 41620 | 32126 | 32126 |
| **MAR_D** | 30125 | 30112 | 27600 | 27543 | 27059 | 27059 | 18813 | 18813 |
| **MAR_T** | 27336 | 27282 | 23737 | 23622 | 22603 | 22601 | 16795 | 16795 |
| **MOU_B** | 38355 | 38305 | 34143 | 34077 | 33928 | 33869 | 25620 | 25620 |
| **MOU_T** | 17129 | 17094 | 15269 | 15104 | 14076 | 14076 | 9999 | 9999 |
| **MTC_B** | 24874 | 24857 | 21735 | 21665 | 21366 | 21364 | 14993 | 14993 |
| **MTC_T** | 26554 | 26543 | 24301 | 24199 | 23072 | 23070 | 17210 | 17210 |
| **NAN_B** | 51480 | 51449 | 47115 | 46975 | 46863 | 46839 | 38312 | 38312 |
| **NAN_T** | 16929 | 16911 | 15223 | 14332 | 13593 | 13593 | 9842 | 9842 |
| **PAR_B** | 48770 | 48706 | 43414 | 43372 | 43128 | 43126 | 33337 | 33337 |
| **PAR_T** | 27870 | 27853 | 25451 | 24999 | 24109 | 24109 | 15961 | 15961 |
| **PEY_B** | 42136 | 42104 | 37801 | 37557 | 36065 | 36064 | 29130 | 29130 |
| **PEY_T** | 37270 | 37129 | 29381 | 29215 | 27188 | 27188 | 20764 | 20764 |
| **POR_B** | 42691 | 42619 | 39115 | 39005 | 38468 | 38468 | 30898 | 30898 |
| **POR_T** | 35385 | 35356 | 32382 | 32315 | 31804 | 31801 | 24469 | 24467 |
| **ROU_B** | 30732 | 30710 | 28002 | 27964 | 26631 | 26631 | 18243 | 18243 |
| **ROU_T** | 26952 | 26916 | 24473 | 23545 | 22833 | 22832 | 16646 | 16646 |
| **ROUM_B** | 32782 | 32754 | 29649 | 29588 | 28963 | 28957 | 21399 | 21399 |
| **ROUM_T** | 14109 | 14006 | 9203 | 9178 | 8693 | 8692 | 5821 | 5821 |
| **SAI_B** | 27519 | 27503 | 25062 | 24605 | 24490 | 24490 | 18906 | 18906 |
| **SAI_T** | 13604 | 13585 | 12266 | 11739 | 11021 | 11021 | 8213 | 8213 |
| **SEV_B** | 40381 | 40339 | 35517 | 35426 | 33335 | 33325 | 23383 | 23383 |
| **SEV_T** | 10530 | 10501 | 9030 | 8993 | 8977 | 8976 | 5790 | 5790 |
| **SOUC_B** | 24161 | 24147 | 22114 | 22056 | 21265 | 21265 | 15823 | 15823 |
| **SOUC_T** | 19759 | 19717 | 17194 | 17157 | 16709 | 16708 | 12377 | 12377 |
| **VAL_B** | 14168 | 14145 | 12307 | 12212 | 12203 | 12168 | 9665 | 9665 |
| **VAL_T** | 18145 | 18129 | 16368 | 16252 | 15268 | 15266 | 10653 | 10653 |
| **VALL_B** | 32212 | 32199 | 29646 | 29548 | 29190 | 29188 | 23101 | 23101 |
| **VALL_T** | 13506 | 13484 | 12200 | 12148 | 11813 | 11813 | 9252 | 9252 |
| **VER_B** | 34985 | 34958 | 31315 | 31242 | 30443 | 30443 | 22147 | 22147 |
| **VER_D** | 31295 | 31258 | 27911 | 27867 | 26439 | 26439 | 18844 | 18844 |
| **VER_T** | 29516 | 29470 | 25749 | 25666 | 23814 | 23814 | 17350 | 17350 |
| **VERT_B** | 23939 | 23923 | 21897 | 21657 | 21465 | 21395 | 15673 | 15671 |
| **VERT_T** | 22012 | 21985 | 19848 | 19814 | 19744 | 19742 | 13253 | 13253 |

**Table S2:** Summary of the total number of OTUs and Reads taxonomically assigned or assigned to a functional trait.

|  | **OTU** | **Equivalent Percentage OTU** | **Number of Reads** | **Equivalent Percentage**  **Number of Reads** |
| --- | --- | --- | --- | --- |
| **Taxonomic Rank** |  |  |  |  |
| **Kingdom** | 2446 | 100 | 1,745,549 | 100 |
| **Class** | 2410 | 99 | 1,741,121 | 99.7 |
| **Subclass** | 1622 | 66 | 1,525,556 | 87 |
| **Order** | 1256 | 51 | 1,282,246 | 73 |
| **Family** | 1126 | 46 | 943,172 | 54 |
| **Genus** | 660 | 27 | 722,244 | 41 |
| **Species** | 523 | 21 | 392,650 | 22 |
|  |  |  |  |  |
| **Functional Traits** | | | | |
| **Foraging Traits** | 1135 | 46 | 1,105,563 | 63 |
| **Limnetic Habitat** | 1234 | 50 | 792,622 | 45 |

**Table S3:** Summary of known physical characteristics and trophic status of the 48 studied lakes (Z_max_=Maximum Depth, SA= Surface Area).

| **Lake Name** | **Lake Code** | **Elevation**  **(m)** | **Z_max_**  **(m)** | **SA**  **(m^2^)** | **Watershed Area (m^2^)** | **Trophic Status** |
| --- | --- | --- | --- | --- | --- | --- |
| Abbaye | ABB | 910 | 19.5 | 82 | 245.8 | MESOTROPHIC |
| Aiguebelette | AIG | 374 | 71 | 545 | 5306.2 | OLIGO-MESOTROPHIC |
| Alate | ALA | 1868 | 10 | 2 | UNKNOWN | ULTRA-OLIGOTROPHIC |
| Arratille | ARA | 2247 | 12 | 16 | 329.9 | OLIGOTROPHIC |
| Aydat | AYD | 825 | 15 | 60.3 | 2551.1 | EUTROPHIC |
| Ayes | AYE | 1694 | 10 | 1.7 | UNKNOWN | OLIGROTROPHIC |
| Balcere | BAL | 1765 | 14 | 4.5 | UNKNOWN | MESOTROPHIC |
| Barroude | BARR | 2355 | 9 | 9.4 | 484 | UNKNOWN |
| Barterand | BAR | 295 | 15 | 21 | 793.1 | MESOTROPHIC |
| Besse | BESS | 245 | 10 | 2 | 163.3 | UNKNOWN |
| Blanchemer | BLA | 984 | 15 | 9 | 208.9 | UNKNOWN |
| Borderes | BOR | 1765 | 18 | 6.5 | UNKNOWN | OLIGOTROPHIC |
| Bourget | BOUR | 231 | 147 | 4396 | 57408 | OLIGO-MESOTROPHIC |
| Chalain | CHA | 490 | 32 | 232 | 3468.4 | MESOTROPHIC |
| Cheserys | CHE | 2135 | 6 | 0.4 | 35.6 | OLIGOTROPHIC |
| Corbeaux | COR | 887 | 27 | 10 | 91.8 | UNKNOWN |
| Cregut | CRE | 900 | 26 | 35.5 | 8999.7 | EUTROPHIC |
| Espingo | ESP | 1882 | 8 | 7.6 | UNKNOWN | OLIGOTROPHIC |
| Etival | ETI | 795 | 10 | 15 | 391.5 | MESOTROPHIC |
| Gentau | GEN | 1950 | 20 | 9.3 | 205 | MESOTROPHIC |
| Gerardmer | GER | 660 | 38 | 116 | 1365 | MESOTROPHIC |
| Godivelle | GOD | 1239 | 44 | 13.8 | 12.3 | OLIGOTROPHIC |
| Gour de Tazenat | GOU | 630 | 66 | 32.9 | 102.8 | OLIGO-MESOTROPHIC |
| Guery | GUE | 1246 | 20 | 26.8 | 790.1 | MESO-EUTROPHIC |
| Ilay | ILA | 778 | 32 | 72 | 165.7 | MESO-EUTROPHIC |
| Isaby | ISA | 1562 | 6 | 6.3 | 755.8 | OLIGOTROPHIC |
| Lagardelle | LAG | 2387 | 27 | 5.8 | UNKNOWN | OLIGOTROPHIC |
| Lamoura | LAM | 1156 | 9 | 3.5 | 1064.8 | UNKNOWN |
| Landie | LAN | 1000 | 21 | 23.9 | 298.4 | MESO-EUTROPHIC |
| Leman | LEM | 372 | 309 | 58100 | 739500 | MESOTROPHIC |
| Longemer | LON | 736 | 34 | 76 | 665.2 | MESOTROPHIC |
| Maix | MAI | 678 | 15 | 1.5 | 17.2 | UNKNOWN |
| Marion | MAR | 50 | 22.8 | 3.8 | 46.3 | UNKNOWN |
| Mont Coua | MTC | 2797 | 10 | 2.43 | 73.5 | OLIGOTROPHIC |
| Mouriscot | MOU | 21 | 10 | 23 | 119 | EUTROPHIC |
| Nantua | NAN | 475 | 43 | 141 | 1639.8 | MESOTROPHIC |
| Parentis | PAR | 19 | 20 | 3502 | 53739.1 | EUTROPHIC |
| Peyrelade | PEY | 1919 | 28 | 9.7 | UNKNOWN | MESOTROPHIC |
| Port Bielh | POR | 2313 | 19 | 16.4 | 234.5 | OLIGOTROPHIC |
| Remoray | REM | 850 | 27 | 85 | 2486.4 | UNKNOWN |
| Roumazet | ROUM | 2163 | 10 | 1.8 | 22.4 | OLIGOTROPHIC |
| Rousses | ROS | 1059 | 18 | 90 | 2143.7 | OLIGO-MESOTROPHIC |
| Saint-Point | SAI | 850 | 42 | 398 | 21598.2 | MESO-EUTROPHIC |
| Serviere | SER | 1200 | 29 | 16.2 | 49.43 | MESOTROPHIC |
| Soucarrane | SOU | 2291 | 10 | 4.4 | 100.57 | OLIGOTROPHIC |
| Val | VAL | 520 | 25 | 64 | 2038.6 | MESOTROPHIC |
| Verdet | VER | 2736 | 12 | 1.9 | 56.2 | OLIGOTROPHIC |
| Vert | VERT | 1266 | 9 | 1.4 | 176.3 | UNKNOWN |

**Table S4:** Data supporting the dating and sampling design of each sediment core. For each lake: the year of coring, the availability of Xrf, ^14^C and radionuclides data, the depth (cm) for 1986 and 1963 ^137^Cs fallouts and stable lead increase/decrease, the selected depth (cm) for top and bottom samples is reported. NC stands for “not clear”. Methods used to determine the depth of top and bottom samples are indicated in the last column where numbers refer to methods described in Supplementary Methods.

| Name | Coring year | XrF | ^14^C | Radionucleides | 1986 ^137^Cs fallout | 1963 ^137^Cs fallout | Onset of stable lead decrease (XrF) | Increase  in stable lead (XrF) | Depth Top | Depth Bottom | Method top-bottom |
| --- | --- | --- | --- | --- | --- | --- | --- | --- | --- | --- | --- |
| Parentis | 2015 | x |  |  |  |  | 21 | 70 | 0-6 | 62-66 | 1/1 |
| Mouriscot | 2015 | x |  |  |  |  | 12 | 45 | 0-4 | 42-44 | 1/1 |
| Marion | 2015 | x |  | 137Cs |  | 49-56 | 49 | 80 | 0-14 | 80-85 | 2/1 |
| Bourget^a^ | 2010 |  |  | 137Cs, 210Pb |  |  |  |  | 0-2 |  | 3/2 |
| Besse | 2015 | x |  |  |  |  | 25.5 | 72 | 0-6 | 68-72 | 1/1 |
| Barterand | 2015 | x |  |  |  |  | 7.5 | 33 | 0-2 | 30-32 | 1/1 |
| Léman^b^ | 2010 |  |  | 137Cs, 210Pb |  |  |  |  | 0-2 |  | 3/2 |
| Aiguebelette | 2015 | x |  |  |  |  | 6 | 21 | 0-2 | 19-21 | 1/1 |
| Nantua | 2015 | x |  | 137Cs, 210Pb | 09-10 | 09-20 | 15.5 | 90 | 0-5 | 84-89 | 3/2 |
| Chalain | 2015 | x |  | 137Cs | 5.5-6 | 11-12 | 8.5 | 20 | 0-4 | 16-18.5 | 2/1 |
| Val | 2015 | x |  |  |  |  | 5 | 23 | 0-3 | 20-23 | 1/1 |
| Tazenat | 2015 | x |  |  |  |  | 5 | 26 | 0-2.5 | 23.5-25.5 | 1/1 |
| Gérardmer^c^ | 2013 | x | x | 137Cs, 210Pb | 04-05 | 07-08 | 7 | 16 | 0-3 | 14-12 | 3/2 |
| Maix | 2015 | x |  | 137Cs | 06-08 | 20-22 | 13 | 62 | 0-4 | 50-55 | 2/1 |
| Longemer | 2013 | x | x | 137Cs, 210Pb | 07-08 | 10-11 | 9.5 | 22 | 0-4 | 16-18 | 3/2 |
| Ilay | 2015 | x |  |  |  |  | 4 | 37 | 0-3 | 30-34 | 1/1 |
| Etival | 2015 | x |  |  |  |  | 9 | 40 | 0-4 | 36-40 | 1/1 |
| Bonlieu | 2013 |  | x | 137Cs | 04-06 | 08-10 | NA | 24 | 0-4 | 22-24 | 2/1 |
| Aydat^d^ | 2015 | x | x | 137Cs, 210Pb |  |  | 33 | 80 | 0-10 | 65-70 | 1/1 |
| Saint-Point^e^ | 2015 | x |  | 137Cs, 210Pb |  |  | 7.5 | 22 | 0-2 | 20-21 | 1/1 |
| Corbeaux | 2015 | x | x | 137Cs | 05-06 | 09-10 | 8.5 | 20 | 0-4 | 18-20 | 2/1 |
| Crégut^f^ | 2015 | x |  |  |  |  | 21 | 42 | 0-5 | 40-42 | 1/1 |
| Abbaye | 2015 | x |  | 137Cs | 10-12 |  | 18 | 50 | 0-5 | 45-47.5 | 2/1 |
| Blanchemer | 2015 | x |  | 137Cs | 12-14 |  | 16 | 48 | 0-3 | 44-48 | 2/1 |
| Landie | 2015 | x |  | 137Cs, 210Pb | NC | 27-30 | 26 | 56 | 0-9 | 47-50 | 3/2 |
| Rousses | 2015 | x |  |  |  |  | 9.5 | 30 | 0-5 | 26-30 | 1/1 |
| Lamoura | 2015 | x |  |  |  |  | 24 | 37 | 0-10 | 32-37 | 1/1 |
| Serviere | 2015 | x |  |  |  |  | 8 | 22 | 0-3.5 | 20-22 | 1/1 |
| Godivelle | 2015 | x |  | 137Cs, 210Pb | 1-1.5 | NC | 3.5 | 18 | 0-2 | 16-18 | 3/2 |
| Guéry | 2015 | x |  | 137Cs | 12-14 | 20-22 | 16 | 74 | 0-5 | 71-74 | 2/1 |
| Vert | 2015 | x |  |  |  |  | 2 | 8 | 0-2 | 6-8 | 1/1 |
| Isaby | 2014 |  | x |  |  |  |  |  | 0-1 | 5-6 | 4/3 |
| Ayes | 2015 |  |  |  |  |  |  |  | 0-1.5 | 5-6.5 | 5/4 |
| Bordères | 2016 | x | x |  |  |  | 2 | 10 | 0-2 | 5-6.5 | 1/1 |
| Balcère | 2013 |  |  |  |  |  |  |  | 0-2 | 5-6.5 | 5/4 |
| Alate | 2016 | x |  |  |  |  | NC | 10 | 0-1.5 | 5-6.5 | 5/1 |
| Espingo | 2016 | x | x |  |  |  | NC | 6.5 | 0-2 | 5-6.5 | 4/3 |
| Peyrelade | 2016 | x | x |  |  |  | 2.5 | 9 | 0-1.5 | 5-6.5 | 1/3 |
| Gentau | 2014 |  | x |  |  |  |  |  | 0-1.5 | 4-5.5 | 4/3 |
| Chéserys | 2013 | x | x |  |  |  | NC | 3.5 | 0-0.5 | 2-2.5 | 4/3 |
| Roumazet | 2016 | x | x |  |  |  | NC | 9 | 0-1.5 | 5-6.5 | 5/3 |
| Arratille | 2013 |  | x |  |  |  |  |  | 0-1.5 | 6-7.5 | 4/3 |
| Soucarrane | 2016 | x |  |  |  |  | NC | 7 | 0-1.5 | 5-6.5 | 4/3 |
| Port Bielh | 2014 |  | x |  |  |  |  |  | 0-1.5 | 2.5-4 | 4/3 |
| Barroude | 2013 |  | x |  |  |  |  |  | 0-1.5 | 6-7.5 | 4/3 |
| Lagardelle | 2016 | x | x |  |  |  | NC | NC | 0-1.5 | 5-6.5 | 4/3 |
| Verdet | 2015 | x |  | 137Cs, 210Pb | NC | 7-7.5 | NC | NC | 0-2 | 19-21 | 3/2 |
| Mont Coua | 2015 | x |  | 137Cs, 210Pb | NC | NC | NC | 9 | 0-1 | 8-9 | 3/2 |

^a^Additional source: Giguet-Covex et al^1^ ;

^b^Additional source: Alric et al^2^ ;

^c^Additional source: Belle et al^3^ ;

^d^Previous studies of Sarazin et al^4^ and Lavrieux et al^5^ indicated a mean sedimentation rate of 0.46 and 0.52 over the last century. Furthermore, two floods events dated at 1907 and 1846 by Lavrieux et al31 has been identified along the 2015 core from XrF logging at 57.5 and 75.5 cm, respectively.

^e^Nedjai et al^6^ indicated a mean sedimentation rate of 0.21 cm.yr-1 over the last century.

^f^A strong change in terrigenous elements (e.g. Ti) that occured at 31 cm along the 2015 core corresponds to the year 1970 when the watershed surface was artificially increased from 1.5 to 86 km².

(1) Giguet-Covex, C. et al. Sedimentological and geochemical records of past trophic state and hypolimnetic anoxia in large, hard-water lake bourget, french alps. J Paleolimnol 43, 171–190 (2010).

(2) Alric, B. et al. Local forcings affect lake zooplankton vulnerability and response to climate warming. Ecology 94, 2767–2780 (2013).

(3) Belle, S., Verneaux, V., Mariet, A.-L. & Millet, L. Impact of eutrophication on the carbon stable-isotopic baseline of benthic invertebrates in two deep soft-water lakes. Freshwater Biology 62, 1105–1115 (2017).

(4) Sarazin, G., Michard, G., Gharib, I. A. & Bernat, M. Sedimentation rate and early diagenesis of particulate organic nitrogen and carbon in Aydat lake (Puy de Dôme, France). Chemical Geology 98, 307–316 (1992).

(5) Lavrieux, M. et al. 6700 yr sedimentary record of climatic and anthropogenic signals in Lake Aydat (french Massif Central): The Holocene (2013) doi:10.1177/0959683613484616.

(6) Nedjai, R., Nguyen-Trung, C. & Messaoud-Nacer, N. Multi-secular lead (Pb) contamination on a regional scale: Comparative analysis of the Grand-Maclu and Saint-Point lakes in the Jura area, france. Journal of Advanced Chemical Engineering 1, 1–10 (2011).


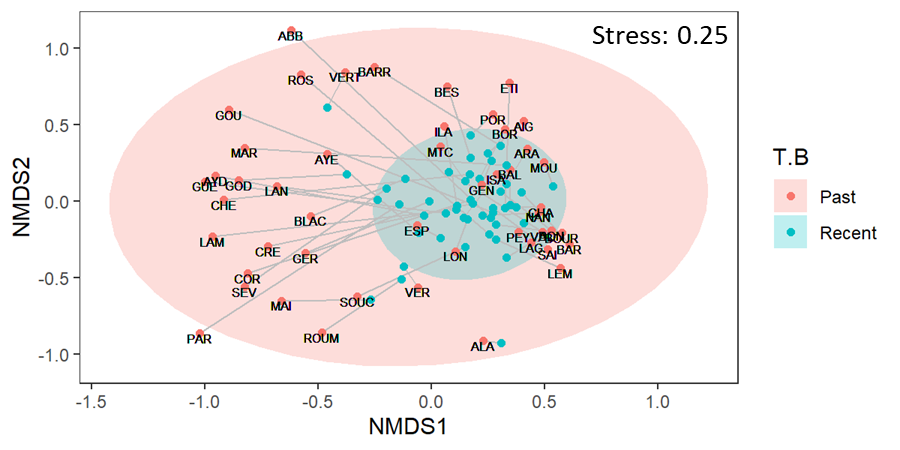


**Figure S1:** NMDS of community compositions of the recent (black dots) and past (purple dots) samples with 95% confidence ellipses represented for each group based on the Bray-Curtis distances of the OTU table. Only the past samples are labelled with their corresponding lake code (cf. Table S1), the gray lines connect recent and past samples from the same lake. Note: some labels are missing to avoid overlapping labels.

**
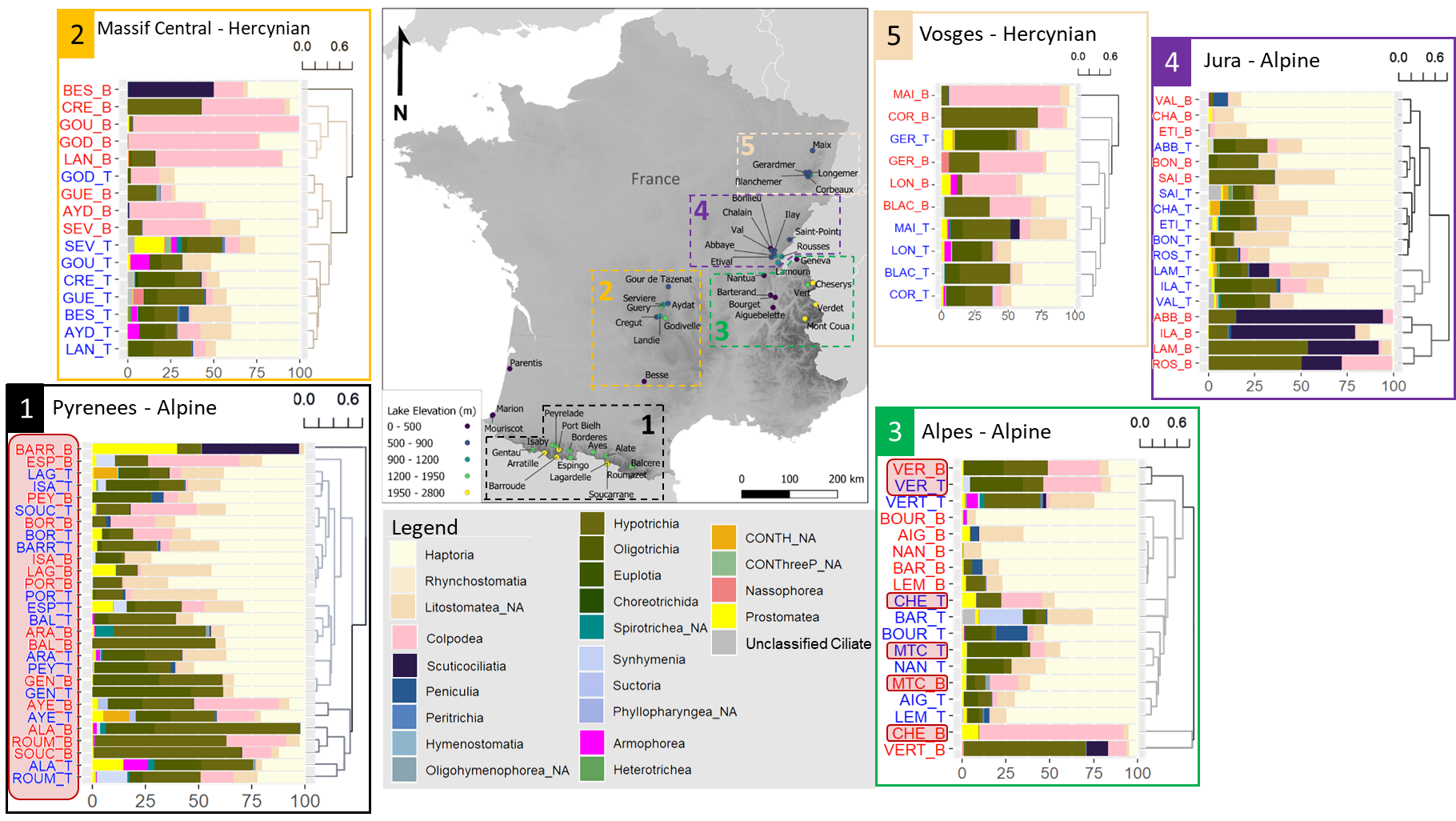
**

**Figure S2:** Dendrograms illustrating the results from the hierarchical cluster analyses applied at the regional level. On the right side of the dendograms the ciliate community compositions are represented as the relative abundance of class and suclass (% of DNA reads). Notes: The samples labels correspond to the lake code (cf. Table S3) followed by “_T” or “_B” indicating the recent (i.e. “top”, in blue) or past (i.e. “bottom”, in red) samples respectively. In the legend, Class_NA was used whenever the Subclass could not be assigned. Lakes located above 1400m in elevation are highlithed in red.


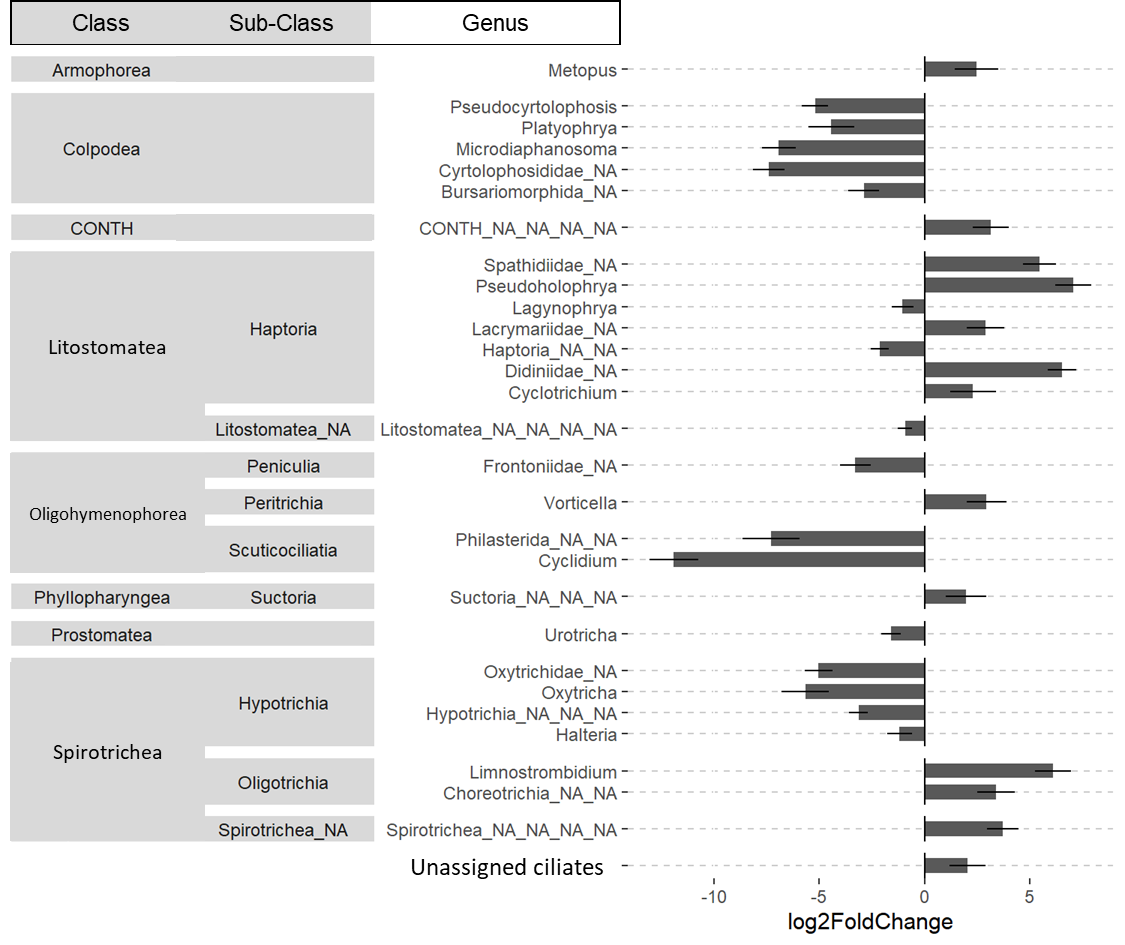


**Figure S3:** Amplitude of change of ciliates community applied at the Genus level between the past and recent strata. Magnitude of change is expressed in log2 fold change, as estimated by the DESeq2 analysis (n= 48 lakes). Only the Genus for which the amplitude of change was significant are presented (two-sided Wald test corrected with the Benjamini and Hochberg method p-value < 0.05). Horizontal lines show the standard error. Any Genus terminating with one “_NA”, two “_NA_NA”, or three “_NA_NA_NA”, corresponds to an assignment at the Familly, Order or Class, respectively (i.e. OTUs for which the assignment stopped at the Class level the pattern “_NA” is repeated three times corresponding to an unknown Order, an unknown Family, and an unknown Genus).


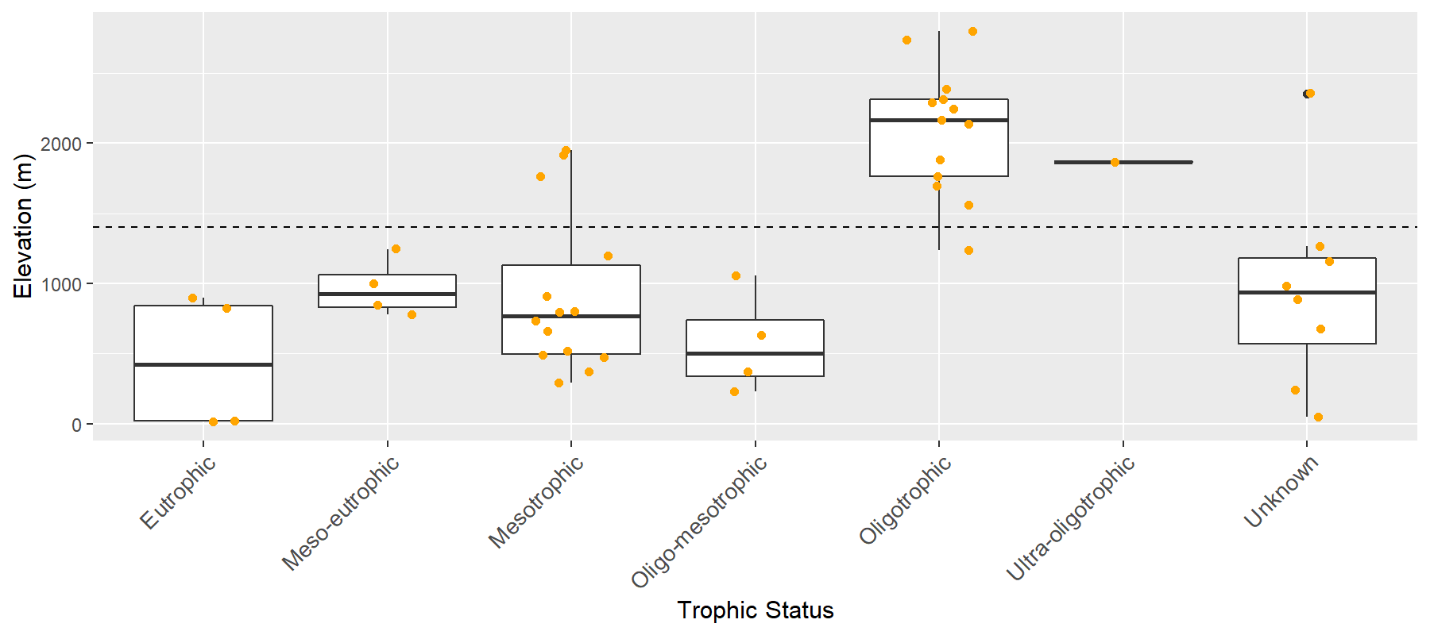


**Figure S4:** Distribution of the elevation gradient among the lake Trophic Status. The dotted line indicates the elevation of 1400 m corresponding to the split identified by the univariate regression tree analysis applied on the Bray-Curtis dissimilarity matrix. The elevation was significantly higher for the oligotrophic lakes than the other trophic status categories (Kruskal-Wallis test: χ^2^=27, df=5, p < 0.05; Post Hoc Wilcoxon-rank test *p_ajusted_* <0.05 for oligotrophic lakes compared to all other trophic status categories; for the pairwise comparison the *p* values were adjusted using the False Discovery Rate approach by Benjamini and Hochberg^1^).

(1) Benjamini Y, Hochberg Y (1995) Controlling the False Discovery Rate: A Practical and Powerful Approach to Multiple Testing. Journal of the Royal Statistical Society: Series B (Methodological) 57:289–300. https://doi.org/10.1111/j.2517-6161.1995.tb02031.x


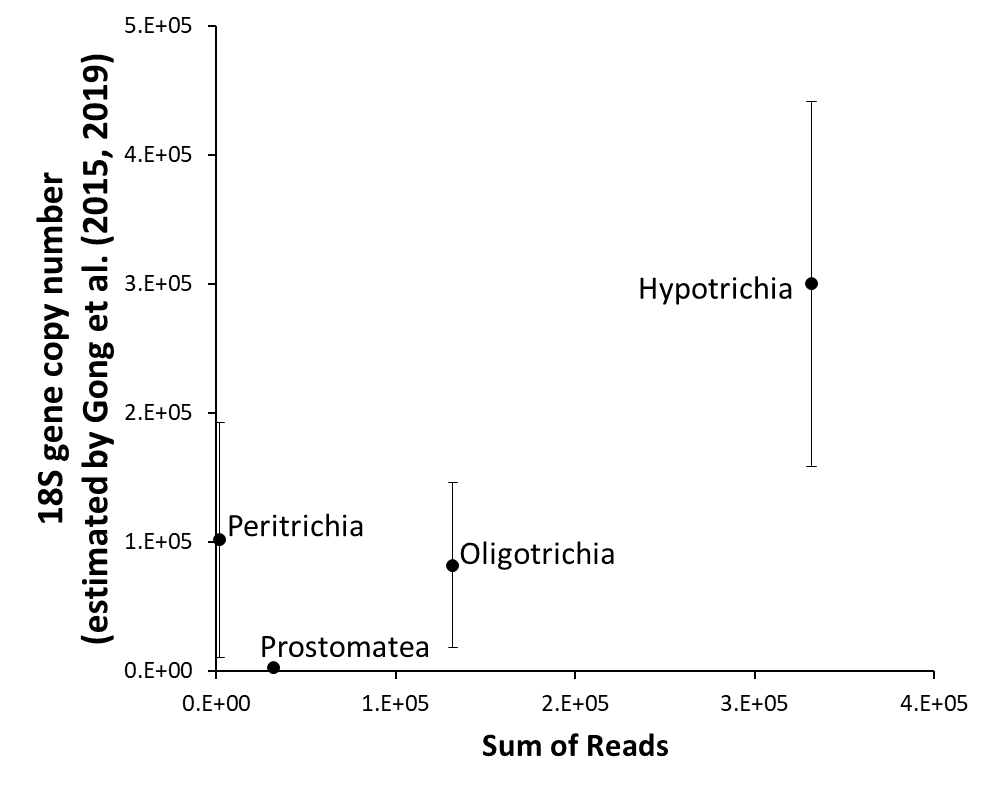


**Figure S5:** Relationship between the number of 18S rRNA gene copies as estimated by Gong et al.^1^ and Gong and Marchetti^2^ and the total number of reads all samples included for the Hypotrichia, Scuticociliatia, Oligohymenomorphorea, Prorodontida and Strombidida.

(1) Gong J, Dong J, Liu X, Massana R (2013) Extremely High Copy Numbers and Polymorphisms of the rDNA Operon Estimated from Single Cell Analysis of Oligotrich and Peritrich Ciliates. Protist 164:369–379. https://doi.org/10.1016/j.protis.2012.11.006

(2) Gong W, Marchetti A (2019) Estimation of 18S Gene Copy Number in Marine Eukaryotic Plankton Using a Next-Generation Sequencing Approach. Front Mar Sci 6:219. https://doi.org/10.3389/fmars.2019.00219
